# Supplementary material for: Causes and Risk Factors of Breast Cancer, What Do We Know for Sure? An Evidence Synthesis of Systematic Reviews and Meta-Analyses
Source: Cancers (Basel). 2024 Apr 20;16(8):1583. doi: 10.3390/cancers16081583 (PMC11049405; doi:10.3390/cancers16081583)
Supplement: Supplementary file 1 [file cancers-16-01583-s001.zip › cancers-2962183-supplementary.pdf]

cancers

MDPI

Supplementary Materials: Causes and Risk Factors of Breast Cancer, What Do We Know for Sure? An Evidence Synthesis of Systematic Reviews and Meta-Analyses

Borghild Løyland *, Ida Hellum Sandbekken, Ellen Karine Grov and Inger Utne

Department of Nursing and Health Promotion, Faculty of Health Sciences, Oslo Metropolitan University, 0130 Oslo, Norway; idahan@oslomet.no (I.H.S.); ellgro@oslomet.no (E.K.G.); inger@oslomet.no (I.U.)
* Correspondence: borglo@oslomet.no

Table S1. External factors.

Author, year, country<br>Methodology	Aim	Period of included studies<br>Number of included studies<br>Number of persons included in the studies	Theme/subjects of the different studies	Quality assessment	Results<br>Risks and causes	Conclusions	Further research	Limitations	CASP scores
**He et al., 2015**<br>Systematic review and Meta-analysis<br>**USA 9 Denmark 3 Island 1 Norway 2 Finland 2 Sweden 2 Israel 1 Germany 1 Australia 1 Japan 1 Singapore 1 Canada 1 China 1 France 1**<br>**Finland, Iceland, Norway and Sweden 1**	To quantitatively evaluate the combined and independent effects of exposure to different sources of circadian disruption on breast cancer risk.	2001-2013<br>28 studies<br>10 Cohort:<br>Total: 1616809. Range: 4036-1148661<br>18 Case- control<br>Total:57108/54320<br>Range: 27/140-34053/18375	Circadian disruption on breast cancer risk.<br>15 studies on shift work, 7 on short sleep duration, 3 on flight attendants, and 6 on light at night were included in the analysis.	NOS Score range from 5-9.<br>MOOSE.	The combined analysis suggested a significantly positive association between circadian disruption and BrCA risk (RR = 1.14; 95% CI 1.08-1.21). Separate analyses showed that the RR for BrCA was 1.19 (95% CI 1.08-1.32) for shift work, 1.120 (95% CI 1.119-1.121) for exposure to light at night, 1.56 (95% CI 1.10-2.21) for employment as a flight attendant, and 0.96 (95% CI 0.86-1.06) for short sleep duration. A dose-response analysis showed that each 10-year increment of shift work was associated with 16% higher risk of BrCA (95% CI 1.06-1.27) based on selected case-control studies. No significant dose-response effects of exposure to light at night and sleep deficiency were found on BrCA risk.	Provides evidence to support a positive association between circadian disruption and breast cancer risk.	Future rigorous prospective studies are needed to confirm these relationships.	Significant heterogeneity from inconsistent definitions of expose across studies.<br>Bias observational studies.<br>Pooled risk estimated should be interpreted with caution.	7/7

**Kamdar et al., 2013**<br><br>Systematic review and Meta-analysis<br><br>**USA 5**<br>**Norway 3**<br>**China 2**<br>**Denmark 1**<br>**Iceland 1**<br>**Germany 1**<br>**Sweden 2**	To evaluate for a general association between night shift work and breast cancer. but also to evaluate whether long- versus short-term exposure to night shift work is associated with greater breast cancer risk.	1996-2011<br><br>15 studies<br><br>5 Cohort:<br>Total:<br>4569/1422189<br>Range: 28/6895-2441/1148661<br><br>10 Case-control:<br>Total: 10635/15716<br>Range: 16/132-6281/6024<br><br>Enrollment period: 1961-2007	Night-shift.<br><br>3 studies with flight attendants, 6 Nurses, and 11 Others.	No quality assessment.	The pooled RR and 95% CIs of breast cancer for individuals with ever night shift work exposure was 1.21 (95% CI, 1.00-1.47, p = 0.056, I^2 = 76%), for short-term night shift workers was 1.13 (95% CI, 0.97-1.32, p = 0.11, I^2 = 79%), and for long-term night shift workers was 1.04 (95% CI, 0.92-1.18, p = 0.51, I^2 = 55%), with substantial between-study heterogeneity observed in all analyses.	Meta-analysis demonstrates there is weak evidence to support the association between night-shift work and risk of breast cancer. Did not support a dose–response relationship of night shift work and breast cancer risk.	Future investigations involving large, diverse occupational, and geographic populations in this area are necessary, given the important public health and policy issues surrounding this issue and the increasing number of around-the-clock workers.	Substantial between-study heterogeneity.<br><br>Methodological limitations of the existing studies.	6/7
**Wang, F. et al., 2013**<br><br>Systematic review and Meta-analysis<br><br>**USA 4**<br>**Norway 2**<br>**Denmark 2**<br>**China 1**<br>**Germany 1**	To conduct a systematic review to sum up evidence of the associations between different aspects of night shift work and female breast cancer.	2001-2012<br><br>10 studies<br><br>3 Cohort: Total: 4510. Range: 717-2441<br><br>7 Case-control: Total: 2593 Range: 132-767<br><br>Missing N control	Night shift.<br><br>5 studies from nursing occupation, 1 military employee, and 4 mixed.	Downs and Black.<br><br>The studies in this review range from 15-19 median 18 Maximum score in this study was 24.	A pooled adjusted RR the association between 'ever exposed to night shiftwork' and breast cancer was1.19 [95% CI 1.05–1.35]. Further meta-analyses on dose–response relationship showed that every 5-year increase of exposure to nightshift work would correspondingly enhance the risk of breast cancer of the female by 3% (pooled RR=1.03, 95% CI1.01–1.05; P heterogeneity <0.001). Our meta-analysis also suggested that an increase in 500-night shifts would result in a 13% RR=1.13, 95% CI1.07–1.21; P heterogeneity=0.06 increase in breast cancer risk.	A positive dose-response relationship is likely to present for breast cancer with increasing years of employment and cumulative shifts involved in the work.	Exploration based on various ethnicities, and research into the mechanisms of how night shift work affects the risk of breast cancer.	Lack of consistent definition of "night shift".<br><br>Cofounding factors.<br><br>The meta-analysis has not adjusted for other risk factors.<br><br>Low case load of each included studies.	7/7
**Jia et al., 2013**<br><br>Systematic review with Meta-analysis<br><br>**Denmark 3**<br>**USA 4**<br>**Norway 2**<br>**France 1**<br>**China 1**<br>**Sweden 1**<br>**Germany 1**	To conduct a systematic review assessing the association between night work and the risk of breast cancer.	1996-2012<br><br>13 studies<br><br>4 Cohort:<br>Total: 4604/167149<br>Range: 94/7436-2441/78562<br><br>9 Case-control:<br>Total:<br>11658/13633<br>Range: 50/259-7035/7035	Night shift.<br><br>4 studies of Female nurses,<br>1 military employee,<br>1 Female radio and telegraph operators, and 7 Others.	NOS with some modification to match the needs of this study.<br><br>The studies in this review range from 3-7.	In the combined analysis of all studies, night work was associated with an increased risk for breast cancer (RR = 1.20, 95% CI = 1.08-1.33). The higher-quality studies showed a similar finding with a pooled RR of 1.40 (95% CI = 1.13-1.73).	The analysis indicates that night work is associated with increased risk of breast cancer.	Additional well-conducted and large-scale epidemiological studies are needed. Recording more precisely and systematically all the most important information about	Limited number of studies.<br><br>Most of the included studies were case–control studies whose designs have limitations in methodology due to selection bias.<br><br>Recall bias.	7/7

							shift work schedules.	Reliability of the information on exposure.	
**Ijaz et al., 2013**<br><br>Systematic review with Meta-analysis<br><br>**USA 4**<br>**Denmark 3**<br>**Norway 3**<br>**Sweden 2**<br>**China 2**<br>**France 1**<br>**Germany 1**	To synthesize the evidence on the potential relationship between nightshift work and breast cancer.	1996-2012<br><br>16 studies<br><br>5 Cohort:<br>Total: 4240/1409911<br>Range: 98/2441-78562/1148661<br><br>11 Case-control:<br>Total: 12907/19001<br>Range: 50/259-6281/6024	Night shift.<br><br>5 studies from Nursing 1 Rad/Telegraph, 1 Military, 1 textile industry, and 8 Others.	GRADE approach for grading the quality of the total evidence.<br><br>Result; Very low quality.	We found an average 5% incremental relative risk increase with 5 years of night shift work. However, cohort studies showed a very small, non-significant risk of 1% as opposed to 9% average in case control studies. Different exposure models or sensitivity analyses did not change these results.	Our findings indicate insufficient evidence for a link between night-shift work and breast cancer.	Objective prospective exposure measurement is needed in future studies.	Did not consider any latency period.<br><br>Under-adjustment for confounding is possible.<br><br>Did not have a real cumulative index in which both duration and intensity of exposure were measured.	7/7
**Qin et al., 2014**<br><br>Systematic review and Meta-analysis<br><br>**USA 2**<br>**Singapore 1**<br>**Australia 1**<br>**Japan 1**<br>**Finland 1**	To gain a greater understanding of the effect of both short and long sleep duration on breast cancer risk.	2005-2013<br><br>6 Studies<br><br>4 Cohort:<br>Total: 147663<br>Range: 12222-77418<br><br>2 Case-control:<br>Total: 12174<br>Range: 2827-9347<br><br>Missing N cases	Sleep duration and breast cancer risk.	NOS Studies ranged from 7-8.<br><br>The scale range from 0-9.	Sort sleep was not associated with breast cancer risk (OR = 1.01; 95% CI = 0.90–1.14; $p = 0.853$; 95% PI = 0.80–1.27) using a random-effects model. Long sleep duration was not associated with breast cancer risk (OR = 0.95; 95% CI = 0.86–1.04; $p = 0.251$; 95% PI = 0.83–1.08) using a random-effects model.	Our study suggested that there is no association between either short or long sleep duration and breast cancer risk.	The association between sleep duration and breast cancer risk and more reasonable criteria for the classification of sleep duration warrant more research.	The sleep duration classification criteria were not consistent among the included studies.<br><br>Few studies.<br><br>The quality of the study was determined by the quality of the individual studies included.	7/7
**Lu et al., 2017**<br><br>Systematic review and Meta-analysis<br><br>**America 5**<br>**China 1**<br>**Singapore 1**<br>**Japan 1**<br>**Australia 1**<br>**Finland 1**	To explore the relationship between sleep duration and breast cancer risk.	2005-2016<br><br>10 studies<br><br>6 Cohort:<br>Total: 368208<br>Range: 12222-110 011<br><br>4 Case-control:<br>Total: 47657<br>Range: 1454-34028<br>(No reported cases, just number of participants)	Sleep and breast cancer risk.	NOS.<br><br>MOOSE.	A J-shaped nonlinear trend was found between sleep duration and breast cancer incidence (*P*non-linear = 0.012); compared with the reference hours (6 h or 7 h), with increasing sleep hours, the risk of breast cancer increased (*P*trend = 0.028). Moreover, a nonlinear relationship was found between sleep duration and estrogen receptor-positive breast cancer (*P*non-linear = 0.013); the risk of estrogen receptor-positive breast cancer increased with	Compared to women with the reference number of sleep hours, women with a longer sleep duration might have a significantly increased risk of breast cancer, especially estrogen receptor-positive breast cancer.	No suggestions for further research.	Misclassification bias.<br><br>The assessment of sleep duration in most studies was based on different time scales.<br><br>Other aspects of sleep such as snoring, sleep quality, and sleep disorder diseases that might	7/7

		Enrollment period: 1975-2012			increasing sleep hours compared to the reference hours (Ptrend = 0.024). However, no nonlinear relationship was found between sleep duration and estrogen receptor-negative breast cancer; the risk of estrogen receptor-negative breast cancer was 1.035 for every additional sleep hour.			influence breast cancer were not considered in the included studies.	
**Yang et al., 2014**<br><br>Systematic review and Meta-analysis<br><br>**USA 8**<br>**Italy 2**<br>**Japan 1**<br>**Singapore 1**<br>**UK 1**<br>**Israel 1**<br>**Finland 1**<br>**Australia 1**	To conduct a dose–response analysis of published observational data to investigate the associations among Light At Night, sleep duration, melatonin levels, and the risk for breast cancer.	2001-2013<br><br>16 studies<br><br>4 Cohort:<br>Total: 5438/147663<br>Range: 143/12222-4233/77418<br><br>12 Case-control:<br>Total: 43074/27493<br>Range: 127/291-34053/14458	Light exposure at night and melatonin.	NOS. Range from 2-9.	High artificial LAN exposure is associated with an increased risk for breast cancer (RR=1.17, 95% CI: 1.11-1.23), but not ambient LAN exposure (RR=0.91, 95% CI: 0.78-1.07). The summary RR for breast cancer is 1.00 (95% CI: 0.995-1.01) for an increment of 1h of sleep per night. No significant dose-response relationship between sleep duration and breast cancer was found either for the linearity test (P_{trend}=0.725) or for the nonlinearity (P_{trend}=0.091) test. An increase in of 15 ng/mg creatinine in urinary 6-sulphatoxymelatonin is associated with a 14% reduced risk for breast cancer (RR=0.86, 95% CI: 0.78-0.95), with a linear dose-response trend (P_{trend}=0.003).	Our study adds to the evidence on the LAN breast cancer theory.	Given the emerging hypothesis that circadian rhythm may play a role in the etiology of breast cancer, as well as other cancers, the difficulty of how best to measure sleep and LAN in epidemiological studies urgently needs to be solved.	Sleep duration and LAN exposure was based on subjective self-reported data among almost all of the individual studies; therefore, misclassification bias cannot be ruled out.<br><br>The inability to fully adjust for various confounders, the observed associations may be confounded by unadjusted risk factors.	7/7
**Liu et al., 2016**<br><br>Systematic review and Meta-analysis<br><br>**Finland, Iceland, Norway and Sweden 1**<br>**Finland 1**<br>**Denmark 1**<br>**Iceland 1**<br>**Norway 1**<br>**USA 3**<br>**Sweden 1**<br>**UK 1**	To conduct an updated meta-analysis to re-assess the risk of breast cancer (BC) among female flight attendants (FFA).	1995-2015<br><br>10 Cohort studies:<br>Total:<br>821/31697<br>Range: 9/287-344/8507<br><br>Enrollment period: 1947-2008	Female flight attendants.	NOS.	The combined SIR (95% CI) for BC in FFAs was 1.40 (95% CI 1.30-1.50), with no significant heterogeneity (P = 0.744; I^2=0.0%) or publication bias (Begg's test: z = 0.72, P = 0.474; Egger's test: t = 0.25, P = 0.805) among the included studies.	Our meta-analysis suggests that FFAs have a higher risk of BC compared with the general population.	More vigorous studies with larger sample sizes based on other populations, including the Chinese, are needed.	Recall and selection bias.<br><br>Confounders.<br><br>The study population varied and limited number of qualified studies.<br>Publication bias.	7/7
**Chen et al., 2010**<br><br>Meta-analysis<br><br>**USA 11**	To consider if the association between exposure to extremely low-frequency electromagnetic fields (ELF-EMF)	2000-2009<br><br>15 Case-control studies:<br>Total:<br>24338/60628	Exposure to extremely low-frequency electromagnetic fields expose.	MOOSE, but not reported.	No significant association between ELF-EMF exposure and female breast cancer risk in total analysis (OR = 0.988, 95% CI = 0.898–1.088) and in all the	Given the possibility of selection bias, exposure misclassification, and the existence of confounding	New state-of-the-art epidemiological studies that incorporate comparable measures	The analyses were restricted by choices and decisions made by the original authors.	5/7

**Norway 2**<br>**Sweden 1**<br>**Canada 1**	and female breast cancer could be confirmed	Range: 99/396-20400/116227<br><br>Enrollment period: 1976-2001	4 studies on Job expose, 6 Magne-tic field measurements, and 5 Use of electric bedding devices.		subgroup analyses by exposure modes, menopausal status, and estrogen receptor status.	variables in the individual studies, it is premature to conclude that the observations reflect a real, rather than artifactual, association.	for both exposure and outcomes are required in order to facilitate future meta-analyses.	The controls were not uniformly defined.	
**Zhao et al., 2014**<br><br>Systematic review and Meta-analysis<br><br>**USA 12**<br>**Sweden 2**<br>**Norway 1**<br>**Canada 1**	To comprehensively analyze the relationship between human exposure to extremely low frequency electromagnetic fields (ELF-EMFs) and breast cancer, and to discuss ELF-EMFs as a potential cause of human breast cancer.	1991-2007<br><br>16 Case-control studies:<br>Total: 44190/131408<br>Range: 63/264-27882/110949<br><br>Enrollment period: 1960-2002	Exposure to extremely low frequency electromagnetic fields (ELF-EMFs).	CASP.	Sixteen research studies outcomes was OR_{DL} = 1.10, 95% CI = (1.01, 1.20), the OR_{MH} of the non-menopause status group was 1.25, 95% CI = (1.05, 1.49), the OR_{MH} of the menopause status group was OR_{MH} = 1.04, 95% CI = (0.93, 1.18).	ELF-EMFs might increasing the risk of human breast cancer. The women's exposure to ELF-EMFs could be the risk factor of breast cancer when they are non-menopausal.	No suggestions for further research.	No limitations in the manuscript.	5/7
**Zhang et al., 2015**<br><br>Systematic review and Meta-analysis<br><br>**USA 14**<br>**Canada 2**<br>**China 2**<br>**Denmark 2**<br>**Mexico 2**<br>**Norway 1**<br>**Japan 1**<br>**Belgium 1**	To evaluate the evidence from observational studies on polychlorinated biphenyl (PCB) exposure and breast cancer risk.	1994-2013<br><br>25 studies<br><br>9 Cohort:<br>Total: 1919/2409<br>Range from 105/112-409/477<br><br>16 Case-control:<br>Total: 4169/4372<br>Range from 60/60-748/659<br><br>Enrollment period: 1973-2011	PCB exposure and breast cancer risk.	NOS.	The results showed that the risk of breast cancer was associated with group II (OR = 1.23, 95% CI: 1.08-1.40) and group III (OR = 1.25, 95% CI: 1.09-1.43) PCBs, but not with group I (OR = 1.10, 95% CI: 0.97-1.24) PCBs or total PCB exposure (OR = 1.09, 95% CI: 0.97-1.22). Group I (potentially estrogenic), group II (potentially anti-estrogenic and immunotoxic, dioxin-like), and group III (phenobarbital, CYP1A and CYP2B inducers, biologically persistent).	Our meta-analysis based on the selected studies found group II and group III PCB exposure might contribute to the risk of breast cancer.	More studies in developing countries as well as studies to explore the relationships between mixtures of organochlorine compounds and breast cancer risk.	One of the included studies is an unpublished thesis.<br><br>Confounders.<br><br>Different PCB exposure measurement.	7/7
**Park et al., 2014**<br><br>Systematic review and Meta-analysis<br><br>**USA 17**<br>**Canada 3**<br>**Belgium 1**<br>**Italy 1**<br>**Denmark 3**<br>**Mexico 2**<br>**Brazil 1**<br>**Spain 1**<br>**Japan 2**	To update a meta-analysis from 2004.	1993-2008<br><br>35 Case-control studies:<br>Total:<br>8160/9401<br>Range: 21/21-748//659<br><br>Enrollment period: 1959-2005	DDE exposure and breast cancer.	MOOSE, but not reported.	The summary odds ratio (OR) for the identified studies was 1.03 (95% confidence interval 0.95-1.12) and the overall heterogeneity in the OR was observed (I^2 = 40.9; p = 0.006). Subgroup meta-analyses indicated no significant association between exposure to DDE and breast cancer risk by the type of design, study years, biological specimen, and geographical region of the study, except from population-based case-control studies with estimated	Existing studies do not support the view that DDE increases the risk of breast cancer in humans. No evidence that there is an association between DDE and breast cancer.	Further studies incorporating more detailed information on DDT exposure and other potential risk factors for breast cancer are needed.	Confounders.<br><br>Missing combined exposures with other chemicals.	6/7

**Sweden 1**<br>**Columbia 1**<br>**Vietnam 1**<br>**Europe 1**					DDE levels in serum published in 1990s.				
**Lin et al., 2013**<br><br>Systematic review and Meta-analysis<br><br>**America 2**<br>**England 1**<br>**Australia 1**<br>**Poland 1**<br>**Sweden 1**<br>**Finland 1**	To assess the relationship between striking life events and primary breast cancer incidence in women.	1995-2009<br><br>7 studies<br><br>3 Cohort:<br>Total: 96604<br>Range: 1462/84334<br><br>4 Case-control:<br>Total 1263/2003<br>Range: 41/78-858/1085	Striking life events.	Downs and Black.	A meta-analysis showed that the pooled OR for striking life events and breast cancer was 1.51 (95% CI 1.15 - 1.97, P = 0.003), indicating that women with striking life events were at 1.5-fold greater risk of developing breast cancer. The pooled OR for severe striking life events and breast cancer was 2.07 (95% CI 1.06 - 4.03), indicating that women with severe striking life events were at 2-fold greater risk of developing breast cancer.	The current meta-analysis showed significant evidence for a positive association between striking life events and primary breast cancer incidence in women.	Further studies are necessary to elucidate the relationship between stress and breast cancer.	Limitations of populations of older people in some cohort studies.<br><br>Selection bias.	7/7
**Santos et al., 2009**<br><br>Systematic review and Meta-analysis<br><br>**Sweden 1**<br>**Finland 1**<br>**Denmark 1**<br>**England 1**<br>**Norway 1**<br>**Australia 2**<br>**USA 1**	To verify the association between stressful life events and primary breast cancer incidence in women.	1986-2003<br><br>8 studies<br><br>2 Cohort:<br>Total 12270<br>Range: 1462-10808<br><br>6 Case-control:<br>Total 6677/ 47665<br>Range: 41/78-4491/44910	Stress and breast cancer risk.	Downs and Black.	Relative risks were: widowhood 1.04 (95% CI: 0.75-1.44; p = 0.800); divorce 1.03 (95%: 0.72-1.48; p = 0.850); and intensity/frequency of stress 1.73 (95% CI: 0.98-3.05; p = 0.059).	Stressful life events as a whole are not associated with risk of breast cancer in women.	Further studies should consider the evaluation of factors associated with breast cancer in women, also in different ethnic populations.	Inclusion of lager studies might have allowed analyzing the studies for sensitivity and possible biases.	7/7
**Chiriac et al., 2018**<br><br>Systematic review<br><br>**USA and Europe 49**<br>**Asia 1**<br>**Australia 2**	To investigate the link between stress and cancer is what compelled this systematic review.	1966-2013<br><br>52 included<br><br>11 Cohort:<br>Total:<br>17166-514958<br>Range: 39/991-11822/315544<br><br>41 Case-control:<br>Total: 1262/217400<br>Range:<br>9/4491-28/141798	Stress and breast cancer risk.	Downs and Black.	We identified 26 positive articles linking personal traits, stressful events and breast cancer, 18 negative articles that did not confirm their hypothesis and 8 articles that could not be classified. Facing heterogeneity, all possible misguiding factors such as: study design, information gathering, stress type, moment of exposure, individual susceptibility and personality, were discussed independently.	Qualitative analysis of articles has revealed a possible association between stress and cancer, especially regarding stressful life events. In the absence of a meta-analysis and taking into account the methodological heterogeneity of the studies, the results are difficult to interpret, and the role of chance is difficult to exclude.	The role of experimental biology studies as a complementary method should possibly be considered in order to measure the relationship between stressful life events and breast cancer development.	A single database.<br><br>Two not accessible in full text.<br><br>Have not conducted a meta-analysis.	6/7

**Bahri, N. et al., 2019**<br><br>Systematic review and Meta-analysis<br><br>**USA 2**<br>**Finland 2**<br>**Australia 1**<br>**UK 2**<br>**Denmark 2**<br>**Sweden 2**	Investigate the relationship between stressful life events and breast cancer.	2000-2016<br><br>11 Cohort studies:<br>Total: 498737<br>Range: 222-345882	Stressful life events.	NOS.	Out of 168 potentially relevant publications, 11 documents met the inclusion criteria. The results showed that history of stressful life events slightly increases the risk of breast cancer [pooled Risk Ratio: 1.11 (95% CI 1.03 to 1.19)].	History of stressful life events could be associated with a moderate increase in the risk of breast cancer. We advise that receiving psychological and counseling services after occurrence of stressful life events of women should be taken seriously.	Conducting more extensive studies considering the perceived stress and assessing the rate of using coping skills is suggested in this field.	No limitations in the manuscript.	7/7

Table S2. Fertility and Drugs.

Author, year, country<br>Methodology	Aim	Period of included studies<br><br>Number of included studies<br><br>Number of persons included in the studies	Theme/subjects of the different studies	Quality assessment	Results<br>Risks and causes	Conclusions	Further research	Limitations	CASP scores
**Zhou et al., 2015**<br><br>Meta-analysis<br><br>**Asia 9**<br>**South America 4**<br>**Europe and Oceania 3**	To detect the prevalence of human papillomavirus (HPV) in breast cancer tissue in patients from northeastern China and define the association between HPV and breast cancer using a meta-analysis.	1999-2012<br><br>16 Case-control studies:<br>Total cases: 2569<br><br>Missing data on each included study	The role of human papillomavirus-infection in breast cancer.	No quality assessment.	An increased risk of breast cancer was observed in association with exposure to HPV (odds ratio [OR] = 3.24, 95% CI = 1.59-6.57), which was influenced by geographic region, HPV DNA source, PCR primer used, and publication period.	HPV, especially high-risk HPV types, may be associated with an increased risk of breast cancer, and this association varies dramatically among geographic regions.	No suggestions for further research.	11 of the studies were not included in the meta-analysis due to limitation of statistical software.<br><br>The PRC based study showed that HVP prevalence in breast cancer was zero.	6/7
**Park et al., 2008**<br><br>Systematic review and Meta-analysis<br><br>**Birth weight and BC-risk:**<br>USA 15<br>Sweden 12<br>Poland 1<br>China 1 Denmark 3<br>UK 2<br><br>**Birth order and BC-risk:**<br>USA 11<br>Sweden 3	To investigate the associations between perinatal factors and subsequent breast cancer risk.	1967-2007<br><br>**Birth weight and BC-risk:**<br>34 studies<br><br>15 Cohort:<br>Total: 9683/525029<br>Range: 37/1024-3140/152590<br><br>19 Case-control:<br>Total: 14 579/32 531<br>Range: 87/87-2386/9801<br><br>**Birth order and BC-risk**:<br>17 studies	Intrauterine environments and breast cancer risk.	No quality assessment.	Heavier birth weights were associated with increased breast cancer risk, with studies involving five categories of birth weight identifying odds ratios (ORs) of 1.24 (95% confidence interval [CI] 1.04 to 1.48) for 4,000 g or more and 1.15 (95% CI 1.04 to 1.26) for 3,500 g to 3,999 g, relative to a birth weight of 2,500 to 2,599 g.<br>Women born to older mothers and twins were also at some increased risk, but the results were heterogeneous across studies and publication years. Birth order, prematurity, and maternal	Our findings provide some support for the hypothesis that in utero exposures reflective of higher endogenous hormone levels could affect risk for development of breast cancer in adulthood.	Additional investigations are needed to determine the extent of any true association of risk with twin status.	A possible misclassification bias for zygosity might have resulted in studies that used sex as a proxy for zygosity.	6/7

Denmark 1<br>Poland 1<br>Nigeria 1<br><br>**Maternal age and BC-risk:**<br>USA 19<br>Sweden 4<br>Poland 1<br>Korea 1 Denmark 3<br>England 1<br>Finland 1<br><br>**Prematurity birth and BC-risk:**<br>USA 8<br>Sweden 6<br>Poland 1<br><br>**Twin status and BC-risk:**<br>USA 7<br>Sweden 2<br>Poland 1<br>Denmark 1<br>USA/England 1<br>Finland 1<br><br>**Maternal or prematernal smoking and BC-risk:**<br>USA 8<br>Poland 1	2 Cohort:<br>Total: 209/1024<br><br>15 Case-control:<br>Total: 17320/42983<br>Range: 24/34-4339/12760<br><br>Missing N on one Cohort study<br><br>**Maternal age and BC-risk**:<br>27 studies<br><br>5 Cohort:<br>Total: 2542/464477<br>Range: 149/1024-1967/384769<br><br>22 Case-control:<br>Total: 28854/54023<br>Range: 153/308-4339/12760<br><br>Missing N on one Cohort, and Sample size on one Case-control Study<br><br>**Prematurity birth and BC-risk:**<br>15 studies<br><br>5 Cohort:<br>Total: 852/13973<br>Range: 12/273-367/5847<br><br>10 Case-control:<br>Total: 10086/29674<br>Range: 87/87-2471/9801<br><br>**Twin status and BC-risk:**<br>13 studies<br><br>5 Cohort:<br>Total: 2734/85860<br>Range: 245/1024-1230/29197<br><br>8 Case-control:<br>Total: 10882/24968<br>Range: 319/768-2522/10052<br><br>**Maternal or prematernal smoking and BC-risk:**	smoking were unrelated to breast cancer risk.

		9 studies<br><br>2 Cohort:<br>Total: 291/5013<br>Range: 42/1024-249/3989<br><br>7 Case-control:<br>Total: 6402/7507<br>Range: 433/53-2497/2380							
**Xu et al., 2009**<br><br>Systematic review and Meta-analysis<br><br>**Sweden 4**<br>**USA 7**<br>**Denmark 2**<br>**Norway 1**<br>**UK 1**<br>**Finland 1**<br>**Poland 1**<br>**China 1**	To investigate the association between birth weight and breast cancer.	1996-2006<br><br>18 studies<br><br>7 Cohort:<br>Total: 6938/3600575<br>Range: 59/2176-3340/3333359<br><br>11 Case-control:<br>Total: 16686/26668<br>Range:<br>89/327-8094/4888	Birth weight as a birth-related factor associated with the risk of breast cancer.	No quality assessment.	Women with their own birth weight >4000 g or 8.5 lb had a higher risk for developing breast cancer than those with birth weight <2500 g or 3000 g (OR = 1.20, 95% CI 1.08-1.34).	Although these results provided no evidence indicating whether birth weight is more strongly related to early-onset than to later-onset breast cancer, our findings suggest an association between birth weight and breast cancer.	Additional studies are warranted in affect modification status.	Confounding variables.<br><br>Publication bias.	5/7
**Guo et al., 2015**<br><br>Systematic review and Meta-analysis<br><br>**USA 7**<br>**Denmark 2**<br>**Europe 2**<br>**China 1**<br>**Sweden 1**<br>**France 1**<br>**Scotland 1**	To evaluate the association between abortion and breast cancer in prospective studies.	1986-2013<br><br>15 Cohort studies<br>Total:<br>31816/2513977<br>Range: 67/390-10246/1529512<br><br>Enrollment period: 1946-2003	Abortion and breast cancer.	MOOSE, but not described the quality assessment.	Non-significant associations of breast cancer with induced abortions (IA) and spontaneous abortions SA were also found among nulliparous women, women with abortion before or after the first full-term pregnancy, women with one or > 2 abortions, and women with first abortion after 30 years old.	The current prospective evidences are not sufficient to support the positive association between abortion (including IA and SA) and breast cancer risk.	Future basic studies are needed to further unveil the etiology of the association between breast cancer and abortion.	Underreported abortion because of stigma.<br><br>Potential publication bias caused by unpublished studies.	6/7
**Kim et al., 2013**<br><br>Meta-analysis<br><br>**USA 12**<br>**Norway 2**<br>**Sweden 2**<br>**Italy 1**<br>**Israel 2**	To analyze the relationship between preeclampsia, pregnancy-induced hypertension (PIH) and maternal risk of breast cancer.	1983-2009<br><br>19 studies<br><br>6 Cohort:<br>Total:<br>17563-1389764<br>Range: 91/2204-5474/689183<br><br>13 Case-control:<br>Total: 17425/26490Range:	Preeclampsia and breast cancer.	No quality assessment.	The pooled estimate of the hazard ratio (HR) associated with preeclampsia was 0.86 (95% CI 0.73-1.01), and that associated with PIH was 0.83 (0.66-1.06), both based on the random effects model.	Some suggestive but not entirely consistent nor conclusive evidence was found on the association between the history of preeclampsia or PIH with the subsequent risk of breast cancer.	Ruther studies are needed to evaluate the effect of different factors on maternal breast cancer risk and prognoses such as population, genetics, treatment	The patients who enrolled in each study could overlap.<br><br>Studies from the same countries over different periods are enrolled.	5/7

		153/337-4668/10052<br><br>Enrollment period: 1942-2004					response and disease severity.	All studies were performed in Europe and US.	
**Sun et al., 2018**<br><br>Meta-analysis<br><br>**USA 2**<br>**Norge 1**<br>**UK 1**<br>**Israel 2**<br>**Danmark 1**<br>**Sverige 2**<br>**Taiwan 1**	To perform an updated meta-analysis of cohort studies to evaluate the association between Preeclampsia (PE), Pregnancy-induced hypertension (PIH), and maternal breast cancer incidence.	2001-2017<br><br>10 Cohort studies:<br>Total: 34273/2417899<br>Range: 74/3804-15856/919712	Preeclampsia, Pregnancy-induced hypertension, and maternal breast cancer incidence.	NOS and MOOSE.	Maternal risk of breast cancer was not significantly affected by PE (risk ration [RR] ¼ 0.93, 95% confidence interval [CI]: 0.82–1.06, p ¼ .27), or PIH (RR ¼ 0.95, 95% CI: 0.81–1.12, p ¼ .54). Interestingly, PE was associated with significantly lowered maternal incidence of breast cancer in women who gave birth to male offspring (RR ¼ 0.79, p < .01), and in those of prospective cohort studies (RR ¼ 0.87, p < .01). However, no significant association between PE and maternal breast cancer was detected in primiparous women, those who gave birth to female offspring, or those of retrospective cohorts.	Current evidence did not support a conclusive association between Preeclampsia, Pregnancy induced hypertension and the maternal risk of breast cancer. Gender of the offspring may influence the association between Preeclampsia and maternal breast cancer incidence.	Further studies are needed to determine whether the preeclamptic pregnancies with male offspring were associated with longer gestational age at birth.	Publication bias.<br><br>Only based on cohort studies.<br><br>Confounding factors.	7/7
**Unar-Munguia et al., 2017**<br><br>Meta-analysis<br><br>**Africa 4**<br>**Asia 28**<br>**Europe 5**<br>**Latin America 5**<br>**USA 23**	To conduct a meta-analysis for breast cancer risk in parous women who breastfed exclusively (or in any mode) versus parous women who formula-fed their infants.	2005-2015<br><br>65 studies<br><br>12 Cohort<br><br>53 Case Control<br><br>Not listed numbers of included in these studies	Breast feeding.	No quality assessment.	The summary relative risk (SRR) for breast cancer in parous women who breastfed exclusively was 0.72, 95% confidence interval (CI) [0.58, 0.90], versus parous women who had never breastfed. For parous women who breastfed in any mode, the SRR was lower in both pre-menopausal women (0.86, 95% CI [0.80, 0.93]) and post-menopausal women (0.89, 95% CI [0.83, 0.95]) The summary dose-response curve was nonlinear (p < .001).	Exclusive breastfeeding among parous women reduces the risk of breast cancer compared with parous women who do not breastfeed exclusively.	Researches need to collect more detailed information of women's breastfeeding mode.	Case-control studies are over-represented and may be biased of our pooled RR.<br><br>Confounding variables.	6/7
**Cohen et al., 2009**<br><br>Systematic review and Meta-analysis<br><br>**USA 5**<br>**Israel 1**<br>**Canada 1**	To determine an association between insufficient milk supply, inability of a mother's breast milk to provide sufficiently for her infant, and breast cancer.	1985-2007<br><br>7 Case-control studies:<br>Total:<br>6601/8466<br>Range:<br>41/17-2603/3145	Insufficient milk supply and breast cancer.	Strobe but did not present the results.	It remains unclear if there is a true association between insufficient milk supply and breast cancer.	Although some studies have shown a strong positive association, there is no consistent evidence for an effect of insufficient milk supply on breast cancer risk.	Exposure definitions are in need of improvement in order to focus on primary insufficient milk supply.	A small sample size.<br><br>Widely varied estimates of the association between insufficient milk supply and breast cancer.	6/7
**Gennari et al., 2015**<br><br>Meta-analysis and qualitative synthesis<br><br>**Israel 7**<br>**USA 3**<br>**Sweden 2**	To assess the potential association between hormonal infertility treatments and breast cancer risk.	1996-2014<br><br>20 Cohort studies:<br>Total: 2347/207914<br>Range: 5/405-497/67608<br><br>Enrollment period: 1960-2011	Hormonal infertility treatments.	MOOSE/ but did not present the results.	No increased risk was detected (SRR = 1.05, 95% CI 0.96-1.14), with a significant heterogeneity (I^2 = 59%, p = 0.001) among studies. In the seven studies with the in vitro fertilization (IVF) procedure, no increase in BC risk was observed (SRR = 0.96, 95% CI 0.80-1.14); in the three NO IVF studies, an	No increased risk was observed in women undergoing IVF. On the other hand, an increase in BC risk cannot be entirely ruled out in women with certain characteristics	No recommendations.	Most of the studies were retrospective and it is difficult to identify closely comparable control groups.	6/7

**Norway 1**<br>**UK 1**<br>**Finland 1**<br>**Australia 2**<br>**Netherlands 1**<br>**Denmark 1**<br>**France 1**					increased BC risk was identified (SRR = 1.26, 95% CI 1.06-1.50). A borderline interaction between type of intervention (IVF vs. NO IVF) and BC risk was observed (p = 0.06). An increased risk with longer follow-up (>10 vs. <10 years) was detected (SRR = 1.13, 95% CI 1.02-1.26 vs. SRR = 0.95, 95% CI 0.85-1.06). Overall, HITs are not associated with an increased BC risk.	(e.g., a specific hereditary make-up) or receiving particular treatment protocols, this must be marginal and/or very rare, because it does not translate into a detectable overall increased risk for the entire population of these women.		Used the general population.	
**Zreik et al., 2010**<br><br>Systematic review and Meta-analysis<br><br>**Israel 8**<br>**USA 5**<br>**Sweden 2**<br>**UK 1**<br>**Italy 1**<br>**Australia 2**<br>**Netherlands 1**<br>**Denmark 1**<br>**France 1**<br>**North America and Europe 1**	To determine the relationship between fertility drugs used in assisted reproductive procedures and the risk of breast cancer.	1995-2009<br><br>23 studies<br><br>15 Cohort:<br>Total:<br>939583 Range: 1082-647704<br><br>8 Case-control:<br>Total 18639/18450<br>Range: 61/120-5564/4794<br><br>Enrollment period 1960-2007<br><br>Missing Cases on Cohort studies	Fertility drugs and breast cancer.	NOS.	The risk of breast cancer was not significantly associated with fertility drug treatment.	The current published data do not suggest higher risk of breast cancer in women who receive fertility treatment, but the lack of long-term follow-up and the inherent weaknesses in some of the published studies have to be cautiously taken into account.	No recommendations.	The follow-up periods were short in some of the studies analyzed in our study.	7/7
**Sergentanis et al., 2014**<br><br>Systematic review and Meta-analysis<br><br>**Israel 4**<br>**Sweden 1**<br>**Finland 1**<br>**Australia 2**	To further pursue and extrapolate our previous effort in the investigation of a potential association between controlled ovarian hyper stimulation (COH) for in vitro fertilization (IVF) and the risk of breast cancer	1999-2013<br><br>8 Cohort studies:<br>Total: 14961/1554332<br>Range: 5/1082-13746/1388371<br><br>Enrollment period: 1978-2011.	Assist of reproductive procedures and the risk of breast cancer.	NOS and PRISMA.	No significant association between IVF and breast cancer was observed either in the group of studies treating the general population (RR = 0.91, 95% confidence interval (CI): 0.74-1.11) or infertile women (RR=1.02, 95% CI: 0.88-1.18), as a reference group.	At present, COH for IVF does not seem to impart increased breast cancer risk. Conclusive statements for the safety of the procedure are reached.	Longer follow-up periods, comparisons versus infertile women, subgroup analyses aiming to trace vulnerable subgroups, adjustment for various confounders and larger informative data sets are needed.	The quality score ranged from 7-9 (9 is max), due to the short follow-up period in seven of the eight studies.	7/7
**Bae & Kim, 2015**<br><br>Systematic Review<br><br>**Korea 6**	To investigate the association between hormone replacement therapy (HRT) and breast cancer risk in Korean women.	2001-2014<br><br>6 studies<br><br>1 Cohort:<br>Total: 39/9579	Hormone replacement therapy (HRT) and breast cancer risk	No quality assessment.	The summary effect size of HRT history from the six articles indicated no statistical significance in breast cancer risk (SOR, 0.983; 95% CI, 0.620 to 1.556).	These facts support no significant effect of HRT history in the risk of breast cancer in Korean women. It is	In order to investigate causality, more patients groups need to be secured.	The research quality is relatively low, as a result of Korean epidemiological research environment.	6/7

		5 Case-control studies:<br>Total: 571/831<br>Range: 48/54-152/304<br><br>Enrollment period: 1995-2008				necessary to conduct a pooled analysis.		Few studies included in this study because of low level of both hormone therapy and breast cancer in Korean.	
**Kim, S. et al., 2018**<br><br>Meta-analysis<br><br>**Korea 1**<br>**France 3**<br>**UK 1**<br>**Denmark 1**<br>**USA 13**<br>**Australian 1**<br>**Japan 1**<br>**Sweden 2**<br>**Taiwan 1**<br>**Europe 1**	To investigate the association between the risk of breast cancer and hormone replacement therapy (HRT).	1994-2016<br><br>25 Cohort studies:<br>Total: 69543/3791688<br>Range: 31/2321-19119/1211238<br><br>One study missing N for cases.	Breast cancer and hormone replacement therapy.	No quality assessment.	Using a random-effects model, HRT use was found to be positively associated with the risk of breast cancer with a pooled hazard ratio (HR) of 1.33 [95% confidence interval (CI) 1.24, 1.44]. Compared with estrogen-only therapy (ET), estrogen-progestin therapy (EPT) was more strongly associated with breast cancer risk. EPT was associated with both ductal and lobular breast cancer risks [for ductal breast cancer, HR=1.51 (95% CI 1.28, 1.78); for lobular breast cancer, HR=1.38 (95% CI 1.20, 1.60)]. According to estrogen receptor (ER) status, all HRTs were associated with the risk of ER-positive breast cancer, but not with that of ER-negative breast cancer.	Asian HRT users had a higher risk of breast cancer than western HRT users. Both ET and EPT were significantly associated with the risk of all breast cancer histological types and ER-positive breast cancer.	Recommend further studies focusing on differences in acquired characteristics as well as genetic and biological differences. The etiologic heterogeneity of breast cancers beyond histological subtype and hormone receptor status also needs to be considered.	Missing subgroup analysis.<br><br>Publication bias.<br><br>Tumor stage was not considered.	6/7
**Samson et al., 2016**<br><br>Systematic review<br><br>**USA 2**<br>**South Africa 1**<br>**Finland 1**<br>**France 1**<br>**Norway/ Sweden 1**	To examine the association between progestin and breast cancer risk.	2000-2007<br><br>6 studies<br><br>3 Cohort:<br>Total:<br>1099/318583<br>Range: 54/73664-880/141892<br><br>3 Case-control:<br>9634/23304<br>Range: 484/2109-4575/11938<br><br>Enrollment period 1990-2000	Progestin and breast cancer risk.	STROBE but did not present the results.	Five of the six studies reported no association between progestin-only formulations (including norethindrone oral contraceptives, depot medroxyprogesterone acetate, injectable, levonorgestrel system users, implantable and intrauterine devices) and breast cancer risk. Duration of use was examined in a few studies with heterogeneous results.	Unlike studies of other oral contraceptives, studies indicate that progestin-only formulations do not increase the risk of breast cancer, although the literature is hampered by small sample sizes.	Future research is needed to corroborate these findings, as further understanding of synthetic progesterone may initiate new prescription practices or guidelines for women's health.	Small samples and sample sizes.<br><br>Various types of progestin.	7/7
**Asi et al., 2016**<br><br>Systematic review and Meta-analysis<br><br>**France 3**	To synthesize the existing evidence about the effect of progesterone in comparison to synthetic progestins, each in combination with estrogens, on the risk of breast cancer and cardiovascular events.	2007-2013<br><br>3 studies<br><br>2 Cohort<br>Total: 1515/85326<br>Range: 16/4949-1499/80377<br><br>1 Case-control:<br>Total:<br>739/816	Progesteron versus synthetic progestin and breast cancer risk	NOS.	Progesterone was associated with lower breast cancer risk compared to synthetic progestins when each is given in combination with estrogen, relative risk 0.67; 95% confidence interval 0.55–0.81.	Observational studies suggest that in menopausal women, estrogen and progesterone use may be associated with lower breast cancer risk compared to synthetic progestin.	More studies are needed to define a potential difference in cardiovascular risk between progesterone and synthetic progestins.	The observational nature of the evidence.<br><br>Small number of studies included.<br><br>Publication bias.<br><br>The results are largely influenced	7/7

		Difficult to find the right N for cases in Cohort						by a single large study.	
**Stute, et al., 2018**<br><br>Systematic review<br><br>**France 10**<br>**USA 2**	To investigate the impact of estrogens combined with micronized progesterone (MP) on the mammary gland, especially on breast density, biopsies (benign breast tissue), and cancer risk.	2002-2017<br><br>12 studies,<br><br>9 Cohort:<br>Total: 11169/356473<br>Range: 8/643-3678/80391<br><br>1 Case-control:<br>Total: 739/816<br><br>2 Systematic reviews:<br>Total: 101356<br>Range: 14475-86881<br><br>Difficult to find BC cases in the Cohort studies, missing in the systematic reviews	Impact of micronized progesterone on breast cancer	No quality assessment.	An international expert panel's recommendations are as follows: (1) estrogens combined with oral (approved) or vaginal (off-label use) micronized progesterone do not increase breast cancer risk for up to 5 years of treatment duration; (2) there is limited evidence that estrogens combined with oral micronized progesterone applied for more than 5 years are associated with an increased breast cancer risk; and (3) counseling on combined menopausal hormone therapy (MHT) should cover breast cancer risk – regardless of the progestogen chosen.	Yet, women should also be counseled on other modifiable and non-modifiable breast cancer risk factors in order to balance the impact of combined MHT on the breast.	No recommendactions.	Compliance, dosage and route of application of MP in E3N and MISSION were not exactly known.<br><br>The E3N report from 2014 did not differentiate between MP and dydrogesterone.<br><br>E3N was the high rates of MHT changes over time.	4/7
**Walker et al., 2011**<br><br>Meta-analysis<br><br>**USA 4**<br>**Europe 2**<br>**Japan 1**	To estimate the effect of a doubling of circulating premenstrual estradiol levels on the risk of breast cancer.	1987-2006<br><br>7 Case control studies:<br>Total: 693/1609<br>Range:<br>17/44-283/551	Estradiol levels.	No quality assessment.	Overall, we found weak evidence of a positive association between circulating E2 levels and the risk of breast cancer, with a doubling of E2 associated with an odds ratio of 1.10 (95% CI: 0.96, 1.27).	Our findings are consistent with the hypothesis of a positive association between pre-menopausal endogenous E2 and breast cancer risk.	Further studies are needed to quantify the association. Repeat measurement in each of the women may be helpful.	No limitation listed.	6/7
**Zhu et al., 2012**<br><br>Meta-analysis<br><br>**Norway 1**<br>**Norway/Sweden 1**<br>**UK 1**<br>**Netherlands 2**<br>**USA 3**<br>**China 2**<br>**France 1**<br>**Korea 1**<br>**Japan 1**	To estimate the association between oral contraceptive (OC) use and breast cancer risk.	1989-2010<br><br>13 Cohort studies:<br>Total<br>11722/ 859894<br>Range: 104/11889-3383/116608<br><br>Enrollment period: 1968-2005	Oral contraceptive use and breast cancer.	No quality assessment, only flow diagram.	The combined relative risk (RR) of breast cancer for ever- compared with never-OC users was 1.08 (95% confidence interval [CI]: 0.99-1.17). Dose-response analysis based on five eligible studies showed that every ten-years' increment of OC use was associated with a significant 14% (95% CI: 1.05-1.23) rise in breast cancer risk.	This meta-analysis provides evidence of a non-significant increase in breast cancer risk associated with ever OC use, but the risk for long-term OC users is significantly greater. The study suggests a borderline association between long-term OC use and risk of breast cancer.	More studies with a long follow-up period are needed to investigate the distinct role thst OC components may have in genesis of breast cancer. The overall risks of OC related outcomes in populations with differing prevalences of health problems should also be investigated.	The latter finding is based on only a limited number of studies.	6/7
**Tio et al., 2014**	To perform a systematic review and quantitative meta-analysis	1991-2014<br><br>49 studies	Folate intake and folate blood levels	No quality assessment.	The meta-analysis of total folate showed no statistically significant association with breast cancer OR of 0.98	Breast cancer does not appear to be associated with folate	Examining in developing countries and	Majority of the included studies from	6/7

Systematic review and Meta-analysis<br><br>**Europe 12**<br>**North America 22**<br>**Asia 10**<br>**Central and South America 3**<br>**Oceania 2**	of observation studies on folate intake and the risk of breast cancer, including analysis of hormone receptor subtypes of breast cancer.	17 Cohort<br>Total: 28829/ 895631<br>Range: 221/11619-3898/90663<br><br>32 Case-control:<br>Total: 21221/ 53176<br>Range: 43/128-2569/5718	and the risk of breast cancer.		(95% CI 0.91-1.07). There was no significant association between either dietary or total folate intake and breast cancer when stratified by hormonal receptor status. The meta-analysis of blood folate levels found no significant association with the risk of breast cancer, with an OR of 0.86 (95% CI 0.60-1.25).	intake, and this did not vary by menopausal status or hormonal receptor status. Folate blood levels also do not appear to be associated with breast cancer risk.	populations with very low folate intake will be needed	developed countries.<br><br>Few studies on blood folate levels.<br><br>Unable to compare risk before and after folate fortifications in the US studies.	
**Zhang, et al., 2014**<br><br>Systematic review and Meta-analysis<br><br>**USA 7**<br>**Sweden 2**<br>**Canada 1**<br>**France 1**<br>**China 1**<br>**Denmark 1**<br>**Australian 1**	To summarize the evidence regarding folate intake and the risk of breast cancer.	1999-2011<br><br>14 studies.<br><br>12 Cohort<br>Total: 17355/623816<br>Range: 71/11699-1411/88818<br><br>2 Case-control:<br>Total: 1205/ 53042<br>Range: 59/24697-191/28345<br><br>Cases is split up into how much daily Folate dose	Folate intake and folate blood levels and the risk of breast cancer.	NOS.	Folate intake had little effect on the breast cancer risk (relative risk (RR) for highest versus lowest category = 0.97; 95% CI, 0.90-1.05; P = 0.451). Dose-response meta-analysis also suggested that a 100 mug/day increase in folate intake had no significant effect on the risk of breast cancer (RR = 0.99; 95% CI, 0.98-1.01; P = 0.361).	Our study revealed that folate intake had little or no effect on the risk of breast cancer; moreover, a dose-response meta-analysis suggested a J-shaped association between folate intake and breast cancer. According to our dose-response meta-analysis, a daily folate intake of 200–320 mg appeared to associate with a lower risk of breast cancer; in contrast, increased breast cancer risk was associated with a daily folate intake 400 mg/d.	Focus on specific populations in order to analyze primary breast cancer prevention.	Publication bias.<br><br>Data regarding breast cancer in premenopausal or postmenopausal women were unavailable.<br><br>The analysis used pooled data (individual data were not available).	7/7
**Takkouche et al., 2008**<br><br>Meta-analysis<br><br>**USA 29**<br>**UK 2**<br>**Canada 3**<br>**Denmark 3**<br>**Europe 1**	To provide a more definitive answer about a possible inverse relationship between the use of nonsteroidal anti-inflammatory drugs and the risk of breast cancer	1980-2008<br><br>38 studies<br><br>22 Cohort:<br>Total: 50572/2568163<br>Range: 14/8818-19934/734899<br><br>16 Case-control:<br>Total: 36849/57579<br>Range: 252/322-7006/14155<br><br>Enrollment period; undisclosed	Use of non-steroidal anti-inflammatory drugs.	MOOSE.	The results of these studies suggest that overall, NSAID use was associated with reduced risk for breast cancer (relative risk [RR] = 0.88, 95% confidence interval [CI] = 0.84 to 0.93). Specific analyses for aspirin (RR = 0.87, 95% CI = 0.82 to 0.92) and ibuprofen (RR = 0.79, 95% CI = 0.64 to 0.97) yielded similar results.	NSAID use is associated with reduced risk for breast cancer.	Future research should include careful evaluation of the molecular mechanisms involved in the relationship between NSAIDs and breast cancer.	Did not take into account possible interactions with other drugs.<br><br>The quality of the individual studies may largely influence the results of the review.<br><br>Confounding factors.	6/7
**Sergentanis et al., 2010**<br><br>Meta-analysis	To examine whether antibiotic use is	2000-2008<br><br>9 studies	Antibiotics use and breast cancer	No quality assessment.	Antibiotic ever-use was associated with slightly elevated breast cancer	Antibiotic use seems to be associated with slightly elevated	Future analyses and meta-analyses on this	Small number of studies.	5/7

**Denmark 1**<br>**USA 2**<br>**UK 2**<br>**Canada 1**<br>**Finland 2**<br>**New Zealand 1**	associated with breast cancer risk.	3 Cohort:<br>Total: 3759930<br>Range: 9461-2130829<br><br>6 Case control: 13769/74620<br>Range from<br>700/700-3708/20000<br><br>Enrollment period: 1981-2003			risk (pooled OR = 1.175, 95% CI: 0.994-1.387).	breast cancer risk. The underlying nature of the association remains elusive, as it may be direct or due to secondary associations, that is, causal or confounding.	important but fiercely contested topic seem warranted.		
**Qu et al., 2013**<br><br>Systematic review and Meta-analysis<br><br>**USA 5**<br>**France 2**<br>**Netherlands 1**<br>**Australia 1**<br>**Sweden 1**	To evaluate the association between high bone mineral density (BMD) and the risk of breast cancer in postmenopausal women.	1996-2011<br><br>10 studies<br><br>7 Cohort:<br>Total: 1533/70045<br>Range: 45/1504-794/37860<br><br>3 Case-control:<br>Total: 356/833<br>Range: 30/150-200/431<br><br>Enrollment period: 1986-1998	Bone mineral density and risk of breast cancer in postmenopausal women	Strobe checklist for cohort studies.	Higher BMD in the hip (RR 1.62; 95% CI: 1.17-2.06) and in the spine (RR 1.82; 95% CI: 1.07-2.57) were associated with a 62 and 82% increased risk of breast cancer. Per SD, increase in hip BMD and spine BMD were also associated with a higher risk of breast cancer (RR for hip BMD 1.20; 95% CI: 1.09-1.31 and RR for spine BMD 1.26; 95% CI: 1.10-1.41).	Higher BMD was found to be associated with a significantly higher risk of breast cancer in post-menopausal women.	Several well designed and stratified cohort studies with adequate control for confounding factors are needed to get a better understanding of the underlying biology of the link between breast cancer and BMD.	Confounding factors.<br><br>Publication bias.<br><br>Study heterogeneity.	7/7
**Chen, J. H. et al., 2019**<br><br>Systematic review and Meta-analysis<br><br>**Germany 1**<br>**Israel 1**<br>**Canada 1**<br>**USA 3**<br>**France 2**<br>**UK 1**<br>**Netherlands 1**	To investigate the evidence from recent epidemiological studies if the relationship between bone mineral density (BMD) and the risk of breast cancer (BC) remains inconsistent.	1996-2017<br><br>10 Cohort studies<br>Total: 1522/81902<br>Range: 45/1380-794/37860	Bone mineral density and breast cancer risk	NOS.	Compared to the participants with the lowest BMD at the lumbar spine, those with the highest BMD had a significantly lower RR for BC (RR =0.75; 95% CI =0.60–0.93; I^2=23.0%). In the subgroup analyses, although the directions of the results were consistent with those of the main findings, not all showed statistical significance. An association was not detected between BMD at the femoral neck or total hip and the risk of BC (RR =0.94; 95% CI =0.66–1.33; I^2=72.5%). Furthermore, the results of the dose–response analysis did not show a significant association between BMD at the lumbar spine, femoral neck, or total hip and the risk of BC.	There is no relationship between BMD and the risk of BC.	More prospective cohort studies are warranted to further investigate this issue.	Cofounding factors.	7/7
**Vishwakarma G. et al., 2019**<br><br>Systematic review and Meta-analysis<br><br>**India 24**	To find pooled estimates of odds ratio (OR) for the corresponding reproductive and other risk factors in Indian women as compared to controls.	1991-2017<br><br>24 case-control:<br>Total: 11481/13221<br><br>Range: 20/50-2101/2255	Reproductive factors	No quality assessment.	statistically significant association between breast cancer and the following reproductive factors: never breastfeed (OR: 3.69; 95% confidence interval [CI]: 1.70, 8.01), menopausal age >50 years (OR: 2.88; 95% CI: 1.85, 3.85), menarche age <13 years (OR: 1.83; 95% CI: 1.34, 2.51), null parity (OR: 1.58;	The results of this meta-analysis are indicative of significant associations between reproductive factors and breast cancer risk, profoundly so among women experiencing	No recommendations.	Only published studies.	6/7

					95% CI: 1.21, 2.06), postmenopause (OR: 1.35; 95% CI: 1.13, 1.62), and age at the 1st pregnancy >25 years (OR: 1.57; 95% CI: 1.37, 1.80). Family history (FH) of breast cancer (OR: 5.33; 95% CI: 2.89, 9.82), obesity (OR: 1.19; 95% CI: 1.00, 1.42), and urban residence (OR: 1.22; 95% CI: 1.03, 1.44) were also found to be significant risk factors.	menopause after the age of 50, women who never breastfeed and FH of breast cancer.			
**Ji, L.W. et al., 2019**<br><br>Systematic review and Meta-analysis<br><br>**Netherlands 1**<br>**Sweden 1**<br>**China 1**<br>**USA 4**<br>**Thailand 1**<br>**Iran 2**	To evaluate the relationship between the age at first use of oral contraceptives (OC) and breast cancer (BC) risk.	1995-2014<br><br>10 studies<br><br>6 Cohort:<br>Total: 6080/ 419397<br>Range: 70/11414-2917/155723<br><br>4 Case-control studies:<br>Total: 2505/ 266908<br>Range: 175/470-1197/264344	Age at first use of oral contraceptives	NOS.	The pooled RR for BC was 1.24 (95% CI: 1.10–1.41), with moderate heterogeneities (I^2=66.5%, P<.001). No significant publication bias was found (P=.584 for Begg test, P=.597 for Egger test). A linear dose–response relationship between the age at first OC use and BC risk was detected (P=.518 for non-linearity).	A significant linear relationship between the age at first OC use and BC risk was confirmed. No further consistent differences are noted in multiple aspects of BC subtypes defined by progesterone or ER status.	Long-term effect of various OC on cancer risk need to be determined by future and ongoing studies.	1 study was excluded for the dose–response meta-analysis for having only two exposure categories.<br><br>Only 3 studies reported BC subtypes.<br><br>2 studies did not report adjusted variables.<br><br>No study reported OC formulation, frequency of administration or menstrual status at onset.<br><br>The threshold of A1stOC that increases BC risk was not assessed.<br><br>Used summary statistics rather than individual Data.	7/7

Table S3. Alcohol and Tobacco.

Author, year, country Methodology	Aim	Period of included studies<br><br>Number of included studies<br><br>Number of persons included in the studies	Theme/subjects of the different studies	Quality assessment	Results Risks and causes	Conclusions	Further research	Limitations	CASP scores

**Macacu et al., 2015**<br><br>Systematic review and Meta-analysis<br><br>**USA/Canada 1**<br>**Canada 6**<br>**Europe 1**<br>**USA 32**<br>**Japan 9**<br>**Thailand 1**<br>**Taiwan 3**<br>**Sweden 5**<br>**Poland 2**<br>**Norway/Sweden 1**<br>**Finland 1**<br>**Australia 2**<br>**Switzerland 1**<br>**Netherlands 3**<br>**China 7**<br>**Norway 2**<br>**Spain 1**<br>**Germany 1**<br>**India 1**<br>**Serbia 1**<br>**USA/Europe 1**<br>**Phillipines 1**<br>**Turkey 1**<br>**UK 1**<br>**Argentina 1**	To conduct a meta-analysis of observational studies on tobacco smoking and breast cancer occurrence.	1984-2015<br><br>86 included studies<br><br>28 Cohort:<br>66 792/25 030 792<br>Range: 67/9846-9822/4187680<br><br>58 Case-control:<br>68 143/1 136 619<br>Range:100/291-6900/906 639	Active smoking and breast cancer.	No quality assessment.<br><br>PRISMA.	For ever-active smoking, in 27 prospective studies, the SRR for breast cancer was 1.10 (95% CI [1.09-1.12]) with no heterogeneity (I^2 = 0%). In 44 retrospective studies, the SRR was 1.08 (95% CI [1.02-1.14]) with high heterogeneity (I^2 = 59%). SRRs for current active smoking were 1.13 (95% CI [1.09-1.17]) in 27 prospective studies and 1.08 (95% CI [0.97-1.20]) in 22 retrospective studies.	As time passes, the evidence accumulates for considering that active tobacco smoking is associated with a modest, but real increase in the risk of breast cancer.	Further studies in never drinkers are recommended.	Limitations are not reported.	6/7
**Chen, C. et al., 2014**<br><br>Systematic review and Meta-analysis<br><br>**China 51**	To shed light on the potential roles of active and passive smoking on the risk of breast cancer among Chinese females.	1992-2013<br><br>51 studies<br><br>3 Cohort:<br>Total: 1180/73858<br>Range: 84/269-718/72 519<br><br>48 Case-control:<br>Total: 16 189/19 109<br>Range:<br>100/100-1 541/1 598<br><br>Enrollment undisclosed	Active and passive smoking.	NOS	Among Chinese females, there was significant association between passive smoking and the risk of breast cancer [odds ratio (OR): 1.62; 95% confidence interval (CI): 1.39-1.85; I^2 = 75.8%, $P < 0.001$; n = 26] but no significant association between active smoking and the risk of breast cancer (OR: 1.04; 95% CI: 0.89-1.20; I^2 = 13.9%, P = 0.248; n = 31). The OR of exposure to husband's smoking and to smoke in the workplace was 1.27 (95% CI: 1.07-1.50) and 1.66 (95% CI: 1.07-2.59), respectively. The OR of light and heavy passive smoking was 1.11 and 1.41, respectively, for women exposed to their husband's smoke (< 20 and > 20 cigarettes per day), and 1.07 and 1.87, respectively, for those exposed to smoke	This study suggests that passive smoking is associated with an increased risk of breast cancer among Chinese females, and the risk seems to increase as passive exposure to smoke increases.	Although the present study revealed non-significant association between active smoking and breast cancer risk, active smoking still warrants attention.	Potential confounding bias caused by other genetic and environmental factors of breast cancer.<br><br>Inadequate overall power.<br><br>A not significant dose-response relationship between the level of passive exposure to smoke and breast cancer risk.	6/7

					in the workplace (< 300 and > 300 min of exposure per day).				
**Sadri et al., 2007**<br><br>Systematic review and Meta-analysis<br><br>**Not reported**	To examine the risk of breast cancer associated with passive and active smoking and to explore risk heterogeneity among studies	1989-2002<br><br>15 Case Control studies:<br>Total: 34 947/57 654<br>Range: 46/34-22 255/40 832<br><br>Enrollment undisclosed	Passive and active smoking.	No quality assessment.	The pooled risk estimate for breast cancer associated with passive smoking among non-smokers was 1.38 (95% confidence interval [CI]; 1.16-1.65). The pooled OR for active smokers was 1.25 (95% CI; 1.11-1.41). Also, the combined OR for passive and active smokers related to breast cancer was 1.30 (95% CI; 1.17-1.45).	Both passive and active smoking equally increase the risk of female breast cancer.	Investigate the effect of passive and active exposure to cigarette smoke based of total lifetime exposure, year smoked by spouse, exposure at home and work, smoking prior to first pregnancy and age at first exposure.	The included studies have small number of cases.<br><br>Recall bias.	4/7
**Miller, et al., 2007**<br><br>Meta-analysis<br><br>**US 10**<br>**UK 1**<br>**Switzerland 1**<br>**China 2**<br>**Germany 1**<br>**Japan 3**<br>**Korea 1**	To evaluate the data and draw conclusions about the association between exposure to environmental tobacco smoke (ETS) and breast cancer.	1984- 2005<br><br>19 studies<br><br>7 Cohort:<br>Total: 5 392/666 030<br>Range: 115/21805-3140/160130<br><br>12 Case-control:<br>Total: 6 297/9 779<br>Missing two studies.<br><br>It is difficult to summarize the number of cases and controls in this study, because of different categorization<br><br>Enrollment period 1975-2000	Exposure to environmental tobacco/passive smoking.	No quality assessment.	The published data indicate an association between ETS and breast cancer in younger primarily pre-menopausal women.	The California Environmental Protection Agency concluded that regular ETS exposure is causally related to breast cancer diagnosed in younger, primarily pre-menopausal women and that the association is not likely explained by bias or confounding.	Full lifetime historical data on ETS exposure. Prospective studies of both active and passive smoking that quantify full lifetime exposures and examine high-risk subpopulations and windows of susceptibility. Studies that examine biologic mechanisms that would explain the similarity of risks seen in active and passive smoking are warranted.	ETS exposure assessment was limited in many, thus decreasing the ability to find evidence of an effect.<br><br>Thus for many of the prospective studies, limited exposure assessment contaminates the referent group with individuals exposed to ETS and biases the relative risk estimates downwards.	5/7
**Chen, Z. et al., 2015**<br><br>Systematic review and Meta-analysis<br><br>**China 8**	To explore the relationship between passive smoking and female breast cancer.	2001-2010<br><br>8 Case-control studies:<br>Total:<br>4542/5114<br>Range: 84/175-1459/1556	Passive smoking in females.	NOS	The results of the meta-analysis indicated that the combined odds ratio (OR) estimate for those who had been exposed to passive smoke from tobacco was 1.67 (95% confidence interval [CI] = 1.27-2.21).	Results suggest a possible association between passive tobacco smoke and female breast cancer in China.	Further verification of the research that utilizes better measures for passive smoking is needed, and a full exploration into the underlying mechanisms is important.	Possibility of selection bias and information bias.	7/7

**Yang Y. et al., 2013**<br><br>Systematic review and Meta-analysis<br><br>**USA 3**<br>**Japan 3**<br>**Korea 1**<br>**UK 1**<br>**Norway/ Sweden 1**<br>**Europe 1**	To evaluate the association between passive smoking and incidence of female breast cancer.	1999-2011<br><br>10 Cohort studies:<br>Total:<br>14831/782534<br>Range:<br>67/9675-3520/224917<br><br>Enrollment period<br>1982-2006	Passive smoking.	NOS	No association between passive smoking and incident of female breast cancer.<br>Compared with the women without exposure to passive smoking, the overall combined RR of breast cancer was 1.01 (95% confidence interval: 0.96 to 1.06, P = 0.73) among women with exposure to passive smoking.	The results suggest that passive smoking may not be associated with increased incidence of breast cancer.	Further studies should concentrate on questionnaire design and a dose-response analysis of passive smoking.	Most studies assessed passive smoking through self-completed questionnaires containing various items.<br><br>The substantial heterogeneity among studies.	7/7
**DeRoo et al., 2011**<br><br>Systematic review and Meta-analysis<br><br>**USA 13**<br>**Canada 3**<br>**Sweden/ Norway 2**<br>**Switzerland 1**<br>**Germany 1**<br>**Poland 1**<br>**Sweden 1**<br>**UK 1**	A meta-analysis of the association between smoking before a first pregnancy and the risk of breast cancer.	1988-2009<br><br>23 studies<br><br>8 Cohort:<br><br>15 Case-control:<br><br>N in each study; not reported<br><br>Enrollment period 1982-2004	Smoking before pregnancy.	No quality assessment.	We found a weak association between smoking before a first pregnancy and breast cancer, with a 10% greater risk observed among women who smoked before their first pregnancy (regardless of whether or not they continued to smoke after the pregnancy) in comparison with women who had never smoked (summary RR = 1.10, 95% CI: 1.07, 1.14).	Despite extensive study, there is little evidence that smoking increases the risk of breast cancer after taking confounders into account.	No suggestions.	The small number of studies (*n* = 5) that specifically examined smoking only before the first pregnancy.<br><br>Publication bias.	6/7
**Chen J.-Y. et al., 2016**<br><br>Systematic review and Meta-analysis<br><br>**Europe 14**<br>**America 8**<br>**Canada 3**<br>**Europe and America 1**	To investigate any potential association between wine and breast cancer risk.	1983-2013<br><br>26 studies<br><br>8 Cohort:<br>Total 9531/539 721<br>Range:<br>66/5048-4140/148030<br><br>18 Case-control:<br>Total:<br>11 618/13 356<br>Range:<br>78/103-2569/2588<br><br>Enrollment period:<br>1960-2009	Wine drinking.	NOS	A 36% increase in breast cancer risk was observed across overall studies based on the highest versus lowest model, with a combined RR of 1.0059 (95% CI 0.97-1.05) in dose-response analysis. However, 5 g/d ethanol from wine seemed to have protective value from our non-linear model.	Our findings indicate that high consumption of wine contributes to breast cancer risk with protection exerted by low doses.	Further investigations are needed for clarification.	Recall bias included the Whites, lacking diversity of race.<br><br>Underestimation of wine consumption.<br><br>Misclassification of wine ingestion.	7/7
**Nagata et al., 2007**<br><br>Systematic review<br><br>**Japan 11**	To review epidemiological studies on alcohol drinking and breast cancer among the Japanese population.	1966-1997<br><br>11 Studies<br><br>3 Cohort:<br>Total: 553/200901	Alcohol and breast-cancer.	No quality assessment.	A significant positive association was observed in one, but another showed nonsignificant inverse association. Out of the eight case-control studies, two studies showed a significantly increased risk among women who drink	We conclude that epidemiologic evidence on the association between alcohol drinking and breast cancer risk remains	Further studies with large samples, including sufficient number of drinkers and with more	Confounding factors, such as smoking.<br><br>Recall bias.	4/7

		Range: 151/22 200-241/142 857<br><br>8 Case-control:<br>Total: 5517/34 563<br>Range: 49/49-1740/15 084<br><br>Enrollment period 1966-1997			daily and who had higher intake of alcohol, respectively.	insufficient in terms of both the number and methodological quality of studies among the Japanese population.	accurate methods for estimating alcohol intake, are needed.		
**Jayasekara et al., 2016**<br><br>Systematic review and Meta-analysis<br><br>**USA 11**<br>**Greece 1**<br>**Sweden 1**<br>**Finland 1**<br>**Canada 1**<br>**Denmark 1**	Cancers of female breast, upper aero-digestive tract (UADT) (oral cavity, pharynx, larynx, oesophagus) and colorectum are causally related to alcohol consumption. To conduct a systematic review and meta-analysis to summarize measured lifetime alcohol consumption or intake over time.	1977-2013<br><br>16 studies:<br><br>3 Cohort:<br>Total: 9 722/199 318<br>Range: 423/23683-7690/91005<br><br>13 Case-control:<br>Total: 26 337/18 045<br>Range: 113/156-6163/8480<br><br>Enrollment period: 1969-2012	Long-term alcohol intake and breast cancer risk.	No quality assessment.	Sixteen articles for breast, 16 for UADT, and 7 for colorectal cancer met the eligibility criteria. We observed a weak non-linear dose–response relationship for breast cancer and positive linear dose–response relationships for UADT and colorectal cancer. The pooled RRs were 1.28 (95% confidence interval, CI: 1.07, 1.52) for breast, 2.83 (95% CI: 1.73, 4.62) for UADT, 4.84 (95% CI: 2.51, 9.32) for oral cavity and pharynx, 2.25 (95% CI: 1.49, 3.42) for larynx, 6.71 (95% CI: 4.21, 10.70) for oesophageal and 1.49 (95% CI: 1.27, 1.74) for colorectal cancer.	Our findings reinforce an association between alcohol intake during lifetime and the occurrence of breast, UADT, and colorectal cancer, but show that measuring lifetime intake may not substantially increase the strength of the associations between alcohol and cancers of the breast, UADT, and colorectum.	No suggestions.	Potential missing studies.<br><br>Heterogeneity between studies.<br><br>Confounding fators.<br><br>Misclassification of alcohol intake.<br><br>Response bias.	6/7

Table S4. Lifestyle, Physical activity, and Body size.

Author, year, country<br>Methodology	Aim	Period of included studies<br><br>Number of included studies<br><br>Number of persons included in the studies	Theme/subjects of the different studies	Quality assessment	Results<br>Risks and causes	Conclusions	Further research	Limitations	CASP scores
**Wu et al., 2013**<br><br>Meta-analysis<br><br>**USA 19**<br>**Japan 2**<br>**Norway 1**<br>**Norway/Sweden 1**<br>**Finland 2**<br>**Denmark 1**<br>**China 1**<br>**France 1**<br>**Canada 1**<br>**Europe 1**<br>**Sweden 1**	To summarize the evidence from prospective studies regarding the association between physical activity and breast cancer risk.	1994-2012<br><br>31 Cohort studies:<br>Total:<br>63 786/ 2 226 836<br>Range: 77/ 7994 -17986/680000<br><br>Enrollment period undisclosed	Physical activity.	No quality assessment.	Dose-response analysis suggested that the risk of breast cancer decreased by 2% ($P < 0.00$) for every 25 metabolic equivalent (MET)-h/week increments in non-occupational physical activity, 3% ($P < 0.00$) for every 10 MET-h/week (roughly equivalent to 4 h/week of walking in 2 miles/h or 1 h/week of running in 6 miles/h) increments in recreational activity, and 5% ($P < 0.00$) for every 2 h/week increments in moderate plus vigorous recreational activity, respectively.	Results from this meta-analysis indicated that physical activity is significantly associated with reduced risk of breast cancer.	No suggestions.	A wide range of definitions of physical activity.	6/7

**Chen, X. et al., 2019**<br><br>Systematic review and Meta-analysis<br><br>**USA 26**<br>**Norway 2**<br>**Finland 2**<br>**USA/Puerto Rico 2**<br>**Sweden 2**<br>**Europa 2**<br>**France 2**<br>**Japan 2**<br>**Netherlands 1**<br>**Denmark 1**<br>**Norway/Sweden 1**<br>**Canada 1**<br>**China 1**	To evaluate and quantify the association between physical activity (PA) and risk of breast cancer.	1994-2017<br><br>45 Cohort studies:<br>Total:<br>88 310/ 3 270 514<br>Range: 46/1566-8034/370029	Physical activity.	NOS.	The overall relative risk (ORR) for breast cancer was 0.87 (95% CI 0.84-0.90). The inverse association was consistent among all subgroup analyses. In subgroup analysis by PA type, the ORR for total activity was 0.87 (95% CI 0.81-0.93), for recreational activity 0.88 (95% CI 0.85-0.91), for occupational activity 0.91 (95% CI 0.84-0.99), and for nonoccupational activity 0.87 (95% CI 0.83-0.92). A linear relationship was found between breast cancer risk and PA(recreational activity and total activity), and the ORR was reduced by 3% (95% CI 0.95-0.99) for every 10 metabolic equivalent of energy hours per week increment in recreational PA and by 2%(95% CI 0.97-0.99) for every 10 metabolic equivalent of energy hours per week increment in total PA.	Our systematic review strengthens the evidence that an inverse correlation existed between PA and breast cancer risk.	It requires further exploration to find the source of heterogeneity.	Possible misreporting PA.<br><br>No individual-level data for study participants (many studies were old).<br><br>The studies didn't have Dose-response analyses of recreational and occupational PA alone.<br><br>Missing subgroup analysis.	7/7
**Neilson et al., 2016**<br><br>Systematic review and Meta-analysis<br><br>**USA 42**<br>**Japan 6**<br>**Norway 1**<br>**Finland 2**<br>**Denmark 1**<br>**China 3**<br>**Canada 5**<br>**Europe 2**<br>**Sweden 3**<br>**Mexico 1**<br>**Global 1**<br>**Puerto Rico/US 1**<br>**Germany 3**<br>**Poland 2**<br>**Australia 2**<br>**Netherland 2 UK 1 Italy 2**<br>**Switzerland 1**	To estimate breast cancer risk associated with high versus low levels of moderate to vigorous recreational activity, separately for pre-menopausal and forpost-menopausal women.	1994-2015<br><br>80 studies<br><br>36 Cohort: Total<br>46 428/1 397 130<br>Range: 46/1806-8034/257805<br><br>44 Case-control:<br>Total: 66 073/111 066<br>Range:<br>75/102-7757/23163<br><br>Enrollment<br>period 1954-2011	Physical activity.	NOS.	Pooled relative risks (RRs, 95% CI) for women with higher versus lower levels of moderate to vigorous recreational activity were RR = 0.80 (0.74-0.87) and RR = 0.79 (0.74-0.84) for pre-menopausal (43 studies) and post-menopausal (58 studies) breast cancer, respectively, with high heterogeneity.	Although risk estimates may be similar for pre-menopausal and post-menopausal breast cancer, subgroup effects may be menopause dependent. Suggest higher versus lower level of moderate activity are associated with an approximately 20-21% lower risk of pre-menopausal and post-menopausal breast cancer.	Investigate the physical activity-breast cancer association across tumor sub types in pre-menopausal women.	The substantial heterogeneity among studies.<br><br>Measurement error.<br><br>Covariate adjustment.<br><br>Clinical heterogeneity.	7/7
**Zhou et al., 2015**<br><br>Meta-analysis<br><br>**USA 7**<br>**Norway 1**	To evaluate the association between sedentary behaviors and the risk of breast cancer.	1993-2014<br><br>21 studies<br><br>7 Cohort:<br>Total: 63356/2580046	Sedentary behaviors.	NOS.	Subgroup analysis showed that the risks of breast cancer for different domains of sedentary behavior were similar, although only occupational behavior showed statistical significance (OR, 1.10; 95% CI, 1.02–	This meta-analysis of observational epidemiologic studies with the most up-to-date evidence indicated that	No suggestions.	Recall and selection bias in the included studies.<br><br>A wide range of definitions of	7/7

**China 2**<br>**Canada 2**<br>**Netherlands 1**<br>**Poland 3**<br>**Sweden 3**<br>**Turkey 1**<br>**India 1**		Range: 351/25624-51520/1940510<br><br>14 Case-control:<br>Total: 19274/45726<br>Range: 233/485-4863/11646<br><br>Enrollment period undisclosed			1.18) and the combined ORs of breast cancer are of borderline significance for sedentary behavior of daily life (OR, 1.10; 95% CI, 1.00–1.20) and sedentary behavior of leisure time (OR, 1.08; 95% CI, 0.98–1.19).	sedentary behavior should be positively associated with an increased risk of breast cancer.		“sedentary” have been used.	
**Cheragi et al., 2012**<br><br>Meta-analysis<br><br>**Country**<br>**Not reported**	To estimate the overall effect of overweight and obesity on breast cancer risk during pre- and post-menopausal period.	1997-2011<br><br>50 studies<br><br>15 Cohort: 2 104 203<br><br>35 Case-control:<br>Total: 71 216<br><br>Case and controls not reported<br><br>Enrollment period undisclosed	Body mass index and breast cancer.	STROBE.	An inverse but non-significant correlation between BMI and breast cancer risk during pre-menopausal period: OR=0.93 (95% CI 0.86, 1.02); RR_i=0.97 (95% CI 0.82, 1.16); and RR_a=0.99 (95% CI 0.94, 1.05), but a direct and significant correlation during post-menopausal period: OR=1.15 (95% CI 1.07, 1.24); RR_i=1.16 (95% CI 1.08, 1.25); and RR_a=0.98 (95% CI 0.88, 1.09).	Body mass index has no significant effect on the incidence of breast cancer during pre-menopausal period. Overweightness and obesity may have a minimal effect on breast cancer, although significant, but really small and not clinically so important	No suggestions.	Selection bias.<br><br>15 studies were not included.<br><br>Potential confounding variables.	5/7
**Xia et al., 2014**<br><br>Meta-analysis<br><br>**USA 18**<br>**Sweden 3**<br>**Norway 1**<br>**Norway/Sweden 1**<br>**Denmark 2**<br>**Korea 1**<br>**China 2**<br>**Netherlands 5**<br>**France 2**	To quantitatively assess the effect of Body Mass Index (BMI) on breast cancer risk.	1997-2014<br><br>37 studies<br><br>Total: 28 684/1 523 780<br>Range: 52/609-4446/287115<br><br>Enrollment period undisclosed	Body mass index.	No quality assessment.	Significant non-linear dose-response ($P < 0.001$) association was identified between BMI and BC risk in post-menopausal women. Individuals with BMI of 25, 30, and 35 kg/m^2 yielded relative risks (RRs) of 1.02 [95% confidence interval (CI): 0.98–1.06], 1.12 (95% CI: 1.01–1.24), and 1.26 (95% CI: 1.07–1.50), respectively, when compared to the mean level of the normal BMI range. However, inverse result though not significant was observed in pre-menopausal women.	This meta-analysis highlighted that obesity contributed to increased BC risk in a nonlinear dose-response manner in post-menopausal women. Further, the analyses indicated a significant non-linear dose-response association between BMI and BC risk in post-menopausal females.	No suggestions.	Complex confounders.<br><br>Insufficient data available in this meta-analysis, for subgroup analysis.<br><br>The methods used restricted the number of studies included.<br><br>Misclassification bias.	5/7
**Chen, Y. et al., 2017**<br><br>Meta-analysis<br><br>**Europa 12**<br>**USA 12**<br>**Asia 6**<br>**Australia 1**	To find out the different effects of BMI on the risk of breast cancer among pre-menopausal and post-menopausal women, and explore the potential factors that influence those associations.	1997-2014<br><br>31 Cohort studies<br>Total: 42,271/3,318,796<br>Range: 115/719-6808/1222630<br><br>Enrollment period: 1961-2008	BMI, pre- and post-menopausal breast cancer risk.	NOS.	The summary relative risks (RRs) were 1.33 (95% CI: 1.20–1.48) and 0.94(95% CI: 0.80–1.11) among post-menopausal and pre-menopausal women, respectively. The dose-response meta-analysis indicated a positive non-linear association between BMI and breast cancer risk among post-menopausal women, and compared to the mean level of the normal BMI category (21.5 kg/m2) the RR in total	In line with previous studies BMI had different effects on pre-menopausal and post-menopausal breast cancer risk. However, contrary to previous studies, a high BMI was not associated with decreased risk in total	Subgroup analyses suggested that the geographical location or genetic factors may influence the relationship between BMI and breast cancer.	BMI was determined mostly by questionnaire investigation or self-reported.<br><br>The adjustments factors of each study were not the same.	7/7

					post-menopausal women were1.03 (95% CI: 1.02–1.05) per 1 kg/m2 increment. However, no statistically significant association among total pre-menopausal women was detected. In subgroup analysis among European pre-menopausal women, the summary RR was 0.79(95% CI: 0.70–0.88). The non-linear relationship showed a negative non-linear association between BMI and breast cancer risk among European pre-menopausal women. When compared to the mean level of the normal BMI category, the RRs were 0.98 (95% CI: 0.96–1.00) per 1 kg/m2 increment, respectively.	pre-menopausal women. More research is needed to better understand these differences.		The categorization of BMI in a number of articles included in this study was not in accordance with the WHO standard.	
**Sexton et al., 2011**<br><br>Literature review<br><br>**USA**	Body size and breast cancer risk in Hispanic and African American women	1979-2010<br><br>11 studies<br><br>1 Cohort:<br>Total: 52080<br><br>10 Case control:<br>Total: 4807/5224<br>Range:<br>127/317-1371/1400<br><br>Enrollment period undisclosed	Body mass index (BMI) or weight gain and the risk of breast cancer.	No quality assessment.	The results were inconsistent in both race/ethnicity groups, with studies reporting positive, inverse, and null results.	There is an urgent need to identify the roles that both obesity and adult weight gain play in the development of breast cancer in these minorities.	Additional studies are needed to provide more understanding of the etiology of this disease and to explain some of the disparities in incidence and mortality.	Scant data.	4/7
**Hidayat et al., 2018**<br><br>Systematic review and Meta-analysis<br><br>**USA 11**<br>**Israel 1**<br>**Canada 1**<br>**Australia 1**<br>**Japan 2**<br>**Netherlands 2**<br>**UK 1**<br>**Finland/Sweden 1**<br>**China 1**<br>**Norway 1**<br>**Denmark 1**<br>**Finland 1**	To evaluate the association between body fatness at a young age (</=30 years), body fatness, gain and the risk of breast cancer.	1989-2016<br><br>24 Cohort studies:<br>Total:<br>33 606/2 000 548<br>Range: 101/719-9660 /947689	Body fatness.	NOS.	Each 5 kg m(-2) increase in BMI was significantly associated with a 14%, 12% and 17% lower risk of breast cancer later in life among all women, pre-menopausal women and post-menopausal women, respectively.	Higher body fatness at a young age may have a protective role in the later development of breast cancer in both pre-menopausal and post-menopausal women. However, our findings suggest that increased body fatness from a young age is positively associated with post-menopausal breast cancer risk.	Further clarification of the biological mechanisms underpinning the association between higher body fatness at a young age and the risk of breast is warranted.	Recall bias in younger age BMI in some of some studies.	6/7
**Zahmatkesh et al., 2017**	To estimate the odds ratio of overweightness and	2008-2014<br><br>8 Case-control studies:	Obesity and overweightness.	STROBE – but this is not a quality	A significant relation was observed between obesity (OR = 1.81, 95% CI = 1.24 - 2.64) and odds of breast cancer.	Meta-analysis results showed a significant relation between	More research in Iran is necessary for elucidating	Inappropriate data in some studies,	4/7

Systematic review and Meta-analysis<br><br>**2017**<br><br>**Iran 8**	obesity as risk factors of breast cancer in studies conducted in Iran.	Total: 33852<br><br>No more information on sample size.		assessment tool.	A significant relation was also observed between overweightness and odds of breast cancer (OR = 1.46, 95% CI = 1.13 - 1.89).	obesity and overweightness with risk of breast cancer in Iranian women and we encouraged them to decrease their weight via physical activity and diet control.	the association between BMI categories and onset of breast cancer during menopause.	Lack of access to data of summaries.<br><br>Not reported data of BMI during the time of menopause.	
**Chen et al., 2016**<br><br>Meta-analysis<br><br>**USA 11**<br>**Italy 1**<br>**Australia 1**<br>**France 1**<br>**Canada 1**<br>**China 1**<br>**Netherland 1**<br>**Europe 1**	To show the evidence for the relationships between abdominal fatness, as measured by waist circumferences (WC) or waist-to hip ratio (WHR), and risks of pre- and post-menopausal breast cancer.	1998-2015<br><br>18 Cohort studies<br>Total: 20602/885261<br>Range:<br>64/253-<br>2111/155723<br><br>Enrollment period undisclosed	Abdominal fatness.	NOS.	When the most fully adjusted RRs were combined, both WC (14 studies, RR per 10-cm increase = 1.06, 95% CI: 1.04–1.09, I^2 = 29.9%) and WHR (15 studies, RR per 0.1-unit increase = 1.07, 95% CI: 1.01–1.14, I^2 = 52.9%) were significantly positively associated with postmenopausal BC, but neither WC (eight studies, RR per 10-cm increase = 1.05, 95% CI: 0.99–1.10, I^2 = 0%) nor WHR (11 studies, RR per 0.1-unit increase = 1.07, 95% CI: 0.95–1.21, I^2 = 59.7%) were associated with premenopausal BC.	Central obesity measured by waist circumferences but not by waist to hip ratio, is associated with modestly increased risks of both pre- and post-menopausal breast cancer independent of general obesity.	No suggestions.	Confounding factors<br><br>Some small studies with null results have been unpublished.<br><br>The included studies were mostly from Europe and the US.	7/7
**Jansen et al., 2014**<br><br>Systematic review<br><br>**Country**<br>**Not reported**	To synthesize the literature on breast size as a risk factor for breast carcinoma by examining studies addressing this question both directly and indirectly	1960-2013<br><br>50 studies<br><br>16 Breast size studies:<br>Total:<br>9548-48596<br>Range: 42/42-2561/32557<br>4 studies missing data<br><br>8 Breast reduction surgery studies:<br>Total: 955- 3808<br>Range: 10/30-443/2174<br><br>2 study questions did not report numbers.	Breast size.	Not reported.	Increasing breast size appears to be a risk factor for breast cancer, and the evidence is stronger for risk reduction with breast reduction, including prophylactic subcutaneous mastectomy at the extreme.	There is direct and indirect evidence that breast size is an important factor in the risk of developing breast cancer. The breast reduction literature indicates that surgery significantly decreases the potential for carcinoma. The decreased incidence of breast cancer among women with cosmetic breast augmentation has also been fairly well established. The subcutaneous mastectomy, essentially a radical reduction mammoplasty, has been shown in large studies to be an	Well-designed prospective studies are required to further assess this risk factor.	The included studies are limited by their retrospective nature<br><br>Imperfect size.<br><br>Measurement techniques.<br><br>Confounding variables.	4/7

						effective method of risk reduction in the prevention of breast cancer.			
**Bae & Kim, 2016**<br><br>Meta-analysis<br><br>**Japan 3**<br>**Korea 2**<br>**Singapore 1**	To investigate the association between breast density in mammography and breast cancer risk in Asian women.	2003-2015<br><br>6 Studies<br><br>1 Cohort:<br>680/23481<br><br>5 Case-control:<br>Total: 1477/3463<br>Range: 71/-223-374/774	Breast density.	Not reported.	In analyzing the subgroups of pre-menopausal versus post-menopausal women, the percent density (PD) index was confirmed to be associated with a significantly elevated risk for breast cancer (sES, 2.21; 95% CI, 1.52 to 3.21; I(2)=50.0%). The RE-DRMR results showed that the risk of breast cancer increased 1.73 times for each 25% increase in PD in post-menopausal women (95% CI, 1.20 to 2.47).	In Asian women, breast cancer risk increased with breast density measured using the PD index, regardless of menopausal status. We propose the further development of a breast cancer risk prediction model based on the application of PD in Asian women.	Future studies need to investigate how breast density affects cancer risk among people of same race who have emigrated to other places.	Only woman who were born and lived in Asia.	6/7
**Nelson, H. et al., 2012**<br><br>Systematic review and Meta-analysis<br><br>**USA 43**<br>**Canada 4**<br>**Italy 4**<br>**Germany 3**<br>**France 2**<br>**Norway 1**<br>**Netherland 1**<br>**10 European counties: 1**<br>**New Zealand 2**<br>**UK 1**<br>**USA & UK 1**<br>**Island 1**<br>**56 Studies from countries all over the world 1**<br>**Norway & Sweden 1**	To determine which factors increase risk for breast cancer in women aged 40 to 49 years and the magnitude of risk for each factor.	1996-2010<br><br>95 studies, 66 to the Meta-analysis<br><br>17 Cohort:<br>Total: 34 446/1 328 260<br>Range: 128/1000-3538/205348<br><br>49 Case-control:<br>Total: 62 463/83 577<br>Range:<br>65/77-15893/24517<br><br>1 study with unknown N<br><br>Enrollment period:<br>Mid1960s-2006	Woman 40-49 years old, and breast cancer risk.	The U.S. Preventive Services Task Force (USPSTF/Task Force).	Extremely dense breasts on mammography or first-degree relatives with breast cancer were associated with at least a 2-fold increase in risk for breast cancer. Prior breast biopsy, second-degree relatives with breast cancer, or heterogeneously dense breasts were associated with a 1.5- to 2.0-fold increased risk; current use of oral contraceptives, nulliparity, and age 30 years or older at first birth were associated with a 1.0- to 1.5-fold increased risk	Extremely dense breasts and first-degree relatives with breast cancer were each associated with at least a 2-fold increase in risk for breast cancer in women aged 40 to 49 years. Identification of these risk factors may be useful for personalized mammography screening.	Nothing recommended.	Studies reported different measures.<br><br>Variation in the degree of adjustment for confounding factors.<br><br>Potential bias.<br><br>Some women outside the targeted age group were included.<br><br>Publication bias and selective reporting.	7/7
**Namiranian et al., 2014**<br><br>Systematic review and Meta-analysis<br><br>**The Mediterranean countries**	To determine most important risk factors for breast cancer in the Eastern Mediterranean Region.	1971-2012<br><br>30 studies<br><br>4 Cohort:<br>Total: 1405/1029<br>Range 16/0-794/885<br><br>26 Case-control:	Factors of breast cancer.	NOS.	The largest ORs were obtained for history of no live birth (2.25; 95% CI: 1.58-3.18), body mass index (BMI) more than 30 (2.21; 95% CI: 1.71-2.36), age at first pregnancy more than 30 years old (1.52; 95% CI: 1.30-1.77), and meat consumption more than three times per week (1.39; 95% CI: 1.03-1.87). The other important predictors	The most important predictors of breast cancer in EMR were history of no live birth, BMI more than 30, age at first pregnancy more than 30 years old, physical	Further studies need to focus on risk factors	Majority of included studies were hospital based.<br><br>Confounders.<br><br>Only articles published in English.	5/7

		Total:6022/7985<br>Range from 43/39-688/996			were higher education and smoking as risk factors, and physical activity and ovulatory stimulating medication as protective factors.	inactivity, and smoking.			
**Namazi et al., 2019**<br><br>Systematic review and Meta-analysis<br><br>**UK 1**<br>**USA 3**<br>**Sweden 2**<br>**Denmark 1**<br>**Australia 1**<br>**Iran 1**<br>**Malaysia 1**<br>**Brasil 1**<br>**Uruguay 1**	To systematically review the association between fat mass (FM) and the risk of breast cancer, and to conduct a meta-analysis, if possible.	2003-2018<br><br>12 studies<br><br>7 Cohort:<br>Total:<br>5323/242053<br>Range: 228/7523-2913/162691<br><br>5 Case-control:<br>Total:<br>1496/2173<br>Range: 31/41-978/1042	Fat mass and breast cancer risk.	NOS.	The pooled RR for the highest versus the lowest FM (%) of cohort studies was 1.44 (95% CI: 1.33, 1.56; I^2: 63.3%, p ¼ 0.008). The overall effect size for adjusted case-control studies showed no significant association (1.49, 95% CI: 0.77, 2.90; I^2: 93.2%; p ¼ 0.001). After stratification by menopause, it was revealed that the association between FM and the risk of breast cancer in post-menopausal women (2.29, 95% CI: 1.12, 4.68; I^2: 92%, p ¼ 0.0001) was significant, while there was no significant association in pre-menopausal women (0.68, 95% CI: 0.18, 2.58; I^2: 81.3%; p ¼ 0.02).	Cohort studies showed that higher FM is positively associated with the risk for breast cancer. However, only case-control studies on post-menopausal women showed a positive link.	More cohort studies are needed to clarify this association. Introduce a certain cut-off point for FM that is linked with breast cancer risk in various societies.	Limited studies and high heterogeneity.<br><br>Lack of sufficient information in most included cohort studies and limited case-control studies.<br><br>All Cohort studies reported the association in post-menopausal women, could not compare the link in both pre- and post-menopausal women.<br><br>Limited studies that reported results for both HRT and non-HRT.<br><br>Different cut points were considered among studies.	7/7
**Chan, D.S.M. et al., 2019**<br><br>Systematic review and Meta-analysis<br><br>**Denmark 3**<br>**Norway 6**<br>**Sweden 14**<br>**Netherlands 4**<br>**International 1**<br>**Taiwan 1**<br>**China 3**<br>**Japan 7**<br>**Australia 4**<br>**France 5**<br>**Korea 2**<br>**Canada 3**<br>**UK 4**<br>**Italy 1**	To systematically review the complex associations between energy balance-related factors and breast cancer risk.	1989-2017<br><br>142 studies<br>Total:<br>125900/8530000	Physical activity, sedentary behavior, adiposity, and weight change.	Some parts of NOS, self-made.	Higher physical activity was inversely associated with both pre- (RR 0.79, CI 0.69–0.91), and post-menopausal breast cancers, whereas increased sitting time was positively associated with post-menopausal breast cancer. Although higher early adult BMI (ages 18–30 years) was inversely associated with pre- and post-menopausal breast cancers, adult weight gain and greater body adiposity increased breast cancer risk in post-menopausal women, and the increased risk was evident for HR+ but not HR− breast cancers, and among never but not current users of post-menopausal hormones. The evidence was less consistent in pre-menopausal women. There were no associations with adult weight gain,	Physical activity reduces breast cancer risk in both pre- and post-menopausal women, whereas factors reflecting energy imbalance influence the risk differently along the life course of the women. Although higher adiposity at early adulthood may reduce pre- and post-menopausal breast cancer risk, weight gain and excessive adiposity later in life increase the risk,	Better understanding on the impact of these factors on pre- and post-menopausal breast cancers and their subtypes along the life course is needed.	Level of misclassification of cancer as pre- or post-menopausal depending on whether the required information was taken at study baseline or cancer diagnosis.<br><br>Measurement error, as frequency, intensity, and type of activity were poorly characterized in most studies.	6/7

**Finland 3**<br>**Europa 7**<br>**Iceland 1**<br>**USA 73**					inverse associations with adult BMI (study baseline) and hip circumference, and non-significant associations with waist circumference and waist-to-hip ratio that were reverted to positive associations on average in studies accounting for BMI. No significant associations were observed for HR-defined pre-menopausal breast cancers.	consistently in post-menopausal women and evidently for HR+ but not for HR− post-menopausal breast cancers. Under precautionary principle, women should aim to be physically active (at least 150 min/week) and follow a lifestyle that leads to healthy body weight (BMI 18.5–24.9 kg/m2) for breast cancer prevention.		Data on pre- and post-menopausal hormone receptor defined breast cancers were limited.	

Table S5. Nutrition.

Author, year, country<br>Methodology	Aim	Period of included studies<br><br>Number of included studies<br><br>Number of persons included in the studies	Theme/subjects of the different studies	Quality assessment	Results<br>Risks and causes	Conclusions	Further research	Limitations	CASP scores
**Li et al., 2014**<br><br>Systematic review and Meta-analysis<br><br>**Korea 2**<br>**China 2**<br>**Japan 1**<br>**Italy 1**<br>**Europe 1**	To clarify the evidence on the association of dietary mushroom intake with breast cancer risk and to quantify its dose-response relationship.	2005-2013<br><br>7 Studies<br><br>2 Cohort:<br>Total: 4777/1748623<br>Range cases: 1072/3505<br><br>5 Case-control:<br>Total: 2313/2387<br>Range cases: 127/479<br><br>Missing N of controls	Mushroom intake and breast cancer risk.	NOS.	The summary RRs of mushroom consumption on breast cancer were 0.96 (95% CI: 0.91-1.00) for pre-menopausal women and 0.94 (95% CI: 0.91-0.97) for post-menopausal women, respectively.	Mushroom intake may be inversely associated with risk of breast cancer.	Mushroom intake and breast cancer risk need to be confirmed with large-scale prospective studies.	Only two of ten included studies have a prospective design.<br><br>Limited information extracted from the studies.<br><br>Many confounders.	7/7
**Fritz et al., 2014**<br><br>Systematic review<br><br>**US 2**<br>**Germany 2**	To assess the impact on risk on primary breast cancer incidence and risk of breast cancer recurrence.	2007-2011<br><br>5 studies<br><br>3 Cohort:<br>4928/53877<br>Range:<br>788/18861-3261/35016	Black cohosh use in women with or at risk of breast cancer.	NOS.<br><br>Cochrane risk of bias tool.	Two observational studies found no association between black cohosh and risk of breast cancer, whereas 2 studies reported significant reductions in risk of primary breast cancer among post-menopausal women (adjusted odds ratio = 0.47, 95% confidence interval = 0.27-0.82), and risk of recurrence	Current evidence does not support an association between black cohosh and increased risk of breast cancer.	Given conflicting but promising results, and apparent safety, further research is warranted.	Retrospective design.<br><br>Variations in the dose of black cohosh.	6/7

		2 Case-control:<br>Total:<br>4411/12581<br>Range:<br>949/2473-3462/10108			(adjusted hazard ratio = 0.75, 95% confidence interval = 0.63-0.89).				
**Nie et al., 2014**<br><br>Systematic review and Meta-analysis<br><br>**USA 8**<br>**Netherlands 2**<br>**Sweden 1**<br>**Germany 1**<br>**Japan 1**<br>**Denmark 1**<br>**Italy 2**	To examine the association between black tea consumption and risk of breast cancer.	1985-2010<br><br>15 Studies<br><br>7 Cohort:<br>Total:<br>12037/2405602<br>Range:<br>342/1376-5272/1715230<br><br>8 Case-control:<br>Total:<br>15047/13816<br>Range:<br>398/480-5882/5399	Black tea consumption and breast cancer.	No quality assessment.	The results showed no association between black tea consumption and breast cancer risk in overall (OR = 0.97; 95% CI = 0.89-1.05).	The association between black tea consumption and breast cancer incidence remains unclear based on the current evidence.	Further well-designed large studies are needed to confirm our result.	Standard of the lowest and highest levels of black tea are not consistent in each study.<br><br>Confounding factors.	6/7
**Si et al., 2014**<br><br>Meta-analysis<br><br>**China 3**<br>**Italy 1**<br>**Japan 3**<br>**USA 2**<br>**Norway 1**<br>**Germany 1**<br>**Europe1**<br>**Uruguay 1**<br>**Europe/America 1**	To conduct a meta-analysis to evaluate the relationship between egg consumption and breast cancer risk.	1995-2009<br><br>13 studies<br><br>5 Cohort:<br>Total: 19280/811555<br>Range:<br>248/7349-20832/351041<br><br>8 Case-control:<br>Total: 13949/37883<br>Range:<br>355/438-3539/19013	Egg consumption and breast cancer.	No quality assessment.	The meta-analysis results showed that egg consumption was associated with increased breast cancer risk (RR 1.04, 95% CI 1.01-1.08).	Egg consumption was associated with increased breast cancer risk among the European, Asian and post-menopausal population and those who consumed >2, <5/week.	More cohort studies and more subgroup analysis are needed.	Only included articles in English.<br><br>Not evaluate the relationship with two eggs daily.<br><br>The categorical standards for egg consuming were confused.	6/7
**Tang et al., 2009**<br><br>Meta-analysis<br><br>**USA 7**<br>**Denmark 2**<br>**Japan 2**<br>**Italy 2**<br>**Norway 1**<br>**France 1**<br>**Israel 1**<br>**Finland 1**<br>**Sweden 1**	To assess the association between coffee consumption and breast cancer risk.	1969-2002<br><br>18 studies<br><br>9 Cohort:<br>Total:<br>9426/360887<br>Range: 51/4301-5272/88055<br><br>9 Case-control:<br>Total: 15824/14231<br>Range: 310/454-2642/5478<br><br>Enrollment period: 1964-2002	Coffee consumption and breast cancer.	No quality assessment.	The combined RR showed a borderline significant influence of highest coffee consumption (RR, 0.95; 95% CI, 0.90-1.00) or an increment of 2 cups/day of coffee consumption (RR, 0.98; 95% CI, 0.96-1.00) on the risk of breast cancer.	Our findings suggest a possible influence of high coffee consumption or an increased coffee consumption on the risk of breast cancer.	Further studies are needed.	Possibilities of confounders.<br><br>Only indexed journals.<br><br>Coffee exposure assessed differently.	6/7

**Guo et al., 2015**<br><br>Meta-analysis<br><br>**USA 6**<br>**USA/Europe 1**<br>**Denmark 1**<br>**China 1**<br>**Netherlands 1**<br>**Sweden 1**<br>**United Kingdom 1**<br>**France 2**<br>**European 1**	To evaluate the association of red and processed meat intake with breast cancer risk.	1996-2014<br><br>14 studies<br><br>Case-control/Cohort: 14<br>Case-control: 31552/1588890<br><br>Range from 53/551-7119/500000<br><br>Enrollment period 1976-2011<br><br>Missing N on 1 study	Red and processed meat and breast cancer risk.	NOS, but not showed in the article.	The summary RRs (95% CI) of breast cancer for the highest versus the lowest categories were 1.10 (1.02, 1.19) for red meat, and 1.08 (1.01, 1.15) for processed meat. The estimated summary RRs (95% CI) were 1.11 (1.05, 1.16) for an increase of 120 g/day of red meat, and 1.09 (1.03, 1.16) for an increase of 50 g/day of processed meat.	Our findings indicate that increased intake of red and processed meat is associated with an increased risk of breast cancer.	Further research with well-designed cohort or interventional studies is needed to confirm the association.	Confounders.<br><br>Misclassification of meat.<br><br>Intake quantities vary.<br><br>Publication bias.	6/7
**Alexander et al., 2010**<br><br>Meta-analysis<br><br>**USA 9**<br>**Netherlands 2**<br>**Denmark 1**<br>**Swedwn 1**<br>**China 1**<br>**Europe 1**<br>**UK 1**<br>**North America/West Europe 1**<br>**Unknown 1**	To conduct a comprehensive meta-analysis of red or processed meat intake and breast cancer risk.	1989-2009<br><br>18 Cohort studies:<br>Total: 43813<br>Range:<br>53-7379<br><br>Enrollment period 1976-2007<br><br>Missing N sample size	Red and processed meat and breast cancer risk.	No quality assessment.	Overall, weak positive summary associations were observed across all meta-analysis models, with the majority being non-statistically significant.	Red meat and processed meat intake does not appear to be independently associated with increasing the risk of breast cancer.	Further investigations of potential effect modifiers, such as analyses by hormone receptor status, may provide valuable insight to potential patterns of associations.	Limitations are not reported.	5/7
**Taylor et al., 2009**<br><br>Meta-analysis<br><br>**USA 4**<br>**Singapore 1**<br>**Uruguay 1**<br>**Germany 1**<br>**USA/Canada 1**<br>**UK 1**<br>**China 1**	To examine the influence of red meat on breast cancer risk among pre-menopausal women.	1986-2007<br><br>10 studies<br><br>3 Cohort:<br>Total: 1945/178336<br>Range:<br>70/33725-1021/90659<br><br>7 Case-control:<br>Total:<br>1921/16415<br>Range: 32/20-15459/14291<br><br>Enrollment period:<br>1957-2003	Red meat and breast cancer risk.	No quality assessment.	The summary relative risk was 1.24 (95% CI 1.08-1.42). Case-control studies had a risk of 1.57 (95% CI 1.23-1.99), while cohort studies had a summary relative risk of 1.11 (95% CI 0.94-1.31).	The findings indicate that red meat intake may carry a different risk profile in pre-menopausal women as opposed to post-menopausal women.	It is important that there be further research looking at red meat and other dietary variables in different populations.	Recall bias.<br><br>Measurement error with FFQ.	6/7
**Anderson, J. et al., 2018**<br><br>Meta-analysis	To assess whether red and processed meat may be risk factors for	2003-2017<br><br>11 Cohort studies:	Red and processed meat	No quality assessment.	The risk was increased in the highest tertile (>9 g/day) of processed meat consumption (adjusted hazard ratio	High consumption of processed meat was associated with higher	No recommendations	Inability to determine whether the associations	6/7

**USA 6**<br>**UK 2**<br>**Sweden 1**<br>**Europe 1**<br>**France 1**	breast cancer due to their iron content, administration of estrogens to cattle or mutagens created during cooking.	Total: 40257/1648994<br>Range: 102/2367-9305/494036	and breast cancer risk.		[HR] 1.21, 95% confidence interval [CI] 1.08e1.35, p Z 0.001). Collation with 10 previous cohort studies provided data on 40,257 incident breast cancers in 1.65 million women. On meta-analysis, processed meat consumption was associated with overall (relative risk [RR] 1.06, 95% CI 1.01e1.11) and post-menopausal (RR 1.09, 95% CI 1.03e1.15), but not pre-menopausal (RR 0.99, 95% CI 0.88e1.10), breast cancer. In UK Biobank and the meta-analysis, red meat consumption was not associated with breast cancer (adjusted HR 0.99 95% CI 0.88e1.12 and RR 1.03, 95% CI 0.99e1.08, respectively).	overall risk of breast cancer; but this association was driven by post-menopausal breast cancer. After taking account of confounding, red meat consumption was not associated with an overall risk of breast cancer either in UK Biobank or the meta-analysis.	for further research.	varied according to the hormonal receptor status of tumours, due to lack of these data in UK Biobank.<br><br>Cofounding factors.	
**Rezaianzadeh et al., 2018**<br><br>Systematic review and Meta-analysis<br><br>**US 8**<br>**US/Canada 1**<br>**China 2**<br>**Uruguay 2**<br>**UK 1**<br>**Singapore 1**<br>**Germany 1**<br>**Europe 1**	To determine the impact of red meat consumption on breast cancer risk in pre-menopausal women.	1991-2015<br><br>17 studies<br><br>8 Cohort:<br>Total: 20585/937330<br>Range<br>70/829-7379/343662<br><br>9 Case-control:<br>Total: 6090/6227<br>Range:<br>122/199- 2386/1703	Red meat consumption and breast cancer risk in pre-menopausal women:	STROBE statement.	Out of the 513 retrieved studies, 17 (9 case-control and 8 cohort) were entered into the meta-analysis. These studies analyzed 26675 cases of breast cancer and over 943557 control or comparison subjects. The results of the random effects meta-analysis indicated a significant association between red meat consumption and breast cancer risk (relative risk: 1.269; 95% confidence interval: 1.117, 1.441; P-value for heterogeneity=0.002). The pooled relative risk was 1.087 (95% confidence interval: 0.999, 1.183) for cohort studies and 1.548 (95% confidence interval: 1.255, 1.909) for case-control studies.	The results of this meta-analysis showed that the women who consumed red meat had an increased risk of breast cancer. Guidelines have placed red meat consumption for breast cancer risk in category B; i.e., no clear harm or benefit. Thus, the results of this meta-analysis indicate the need to revise the guidelines.	Further studies are required to investigate this association.	Recall bias.<br><br>Using the food frequency questionnaire could result in bias due to measurement error and misclassification in exposure.	7/7
**Farvid et al., 2018**<br><br>Systematic review and Meta-analysis<br><br>**Denmark 1**<br>**USA 8**<br>**Canada 1**<br>**France 2**<br>**Sweden 2**<br>**UK 2**<br>**Japan 1**<br>**Europe 1**<br>**Netherlands 2**	To summarize the evidence regarding the relation of red meat and processed meat consumption with breast cancer incidence	1989-2018<br><br>20 studies<br><br>16 Cohort:<br>Total: 39547/1323013<br>Range: 102/1598-9305/319826<br><br>4 Cases-control: Total: 1355/3020<br>Range: 180/264-579/1560	Red and processed meat consumption and breast cancer incidence.	MOOSE.	Red meat (unprocessed) consumption was associated with a 6% higher breast cancer risk (pooled RR,1.06; 95% confidence intervals (95% CI):0.99-1.14; I^2 = 56.3%), and processed meat consumption was associated with a 9% higher breast cancer risk (pooled RR, 1.09; 95% CI, 1.03-1.16; I^2 = 44.4%).	In the prospective observational studies, high processed meat consumption was associated with increased breast cancer risk. However, red meat was not a significant cause of breast cancer.	Further studies examining molecular subtypes of breast cancer are needed.	Publication bias.<br><br>Residual confounding factors.<br><br>The under- or over-reporting of the amount of food groups could cause measurement error.	7/7

**Chan et al., 2011**<br><br>Meta-analysis<br><br>**Country**<br>**Not reported**	To perform a meta-analysis of cohort and case-control studies that evaluate multivitamin intake and its relationship with breast cancer risk.	2001-2010<br><br>8 studies<br><br>5 Cohort:<br>Total: 12507/347751<br>Range: 743/35023-9619/161806<br><br>3 Case-control:<br>Total:<br>7283/7246<br>Range:<br>861/790-3454/3474	Multivitamin intake and its relationship with breast cancer risk.	Grade Quality of evidence.<br><br>Revman, risk of bias.	Multivitamin use was not significantly associated with the risk of breast cancer.	Multivitamin use is likely not associated with a significant increased or decreased risk of breast cancer.	The results highlight the need for more case-control studies or randomized controlled clinical trials to further examine this relationship.	Recall bias.<br><br>Variation of duration or fervency of use of multivitamin.<br><br>No report of confounding factors.	7/7
**Hidayat, et al., 2016**<br><br>Systematic review and Meta-analysis<br><br>**USA 5**<br>**Finland 1**<br>**France 1**<br>**Norway 1**<br>**Sweden 1**<br>**2Europe 1**<br>**China/**<br>**Singapore 1**	To address the relation between Calcium (Ca) and breast cancer risk.	2002-2013<br><br>11 Cohort Studies:<br>Total: 27962/929654<br>Range:<br>88/3627-7760/319985	Calcium and breast cancer risk.	NOS.<br><br>PRISMA.	Dose-response analysis revealed that each 300 mg/d increase in Ca intake was associated with 2% (RR 0.98; 95% CI 0.96, 0.99), 8% (RR 0.92; 95% CI 0.87, 0.98) and 2% (RR 0.98; 95% CI 0.97, 0.99) reduction in the risk of total, pre-menopausal and post-menopausal breast cancer, respectively.	Our findings suggest an inverse dose-response association between Ca intake and risk of breast cancer.	Additional large prospective studies focusing on the influence of hormone receptor status.	Confounding factors in included studies.<br><br>Inter-relationship between Ca intake and vitamin D intake.<br><br>Moderate heterogeneity across studies.	7/7
**Xie et al., 2013**<br><br>Meta-analysis<br><br>**China 5**<br>**Singapore 1**<br>**Singap/China 1**<br>**Japan 3**<br>**Korea 2**<br>**USA 2**<br>**England 2**<br>**Asia/American 1**<br>**Netherlands 1**<br>**Germany 1**<br>**France 1**<br>**Sweden 1**<br>**Canada 1**	To conduct a meta-analysis on the association between isoflavone intake and breast cancer risk by comprehensively assessing isoflavone exposure in the targeted populations.	1992-2010<br><br>22 studies<br><br>7 Cohort:<br>Total: 3681/254617<br>Range:<br>179/15555-1014/73223<br><br>15 Case-control:<br>Total:<br>12246/14871<br>Range: 167/360-3063/3430	Isoflavone intake and breast cancer risk.	No quality assessment.	Overall, the results showed that isoflavone reduced the breast cancer risk (a combined RR/OR of 0.68, 95% CI: 0.52-0.89) in Asian populations rather than Western populations (a combined RR/OR of 0.98, 95% CI: 0.87, 1.11) for the high-dose category. No significant difference in the studies of Western populations.	Exposure to high isoflavone may be associated with a reduced breast cancer risk in Asian populations, especially in post-menopausal women.	More high-quality rigorous studies, especially long term follow up studies of the association between isoflavone intake and breast cancer should be performed to confirm our result.	Significant heterogeneity is detected among trials.<br><br>Isoflavone consumption is assessed in a small number of studies.<br><br>Confounders as other lifestyle, more fruit, and less alcohol.	6/7
**Mourouti et al., 2015**<br><br>Systematic review<br><br>**Soy product and Isoflavones:**	To report in a systematic way the current scientific evidence relating breast cancer and diet	2002-2012<br><br>**25 Alcohol** studies<br>20 Cohort:<br>Total: 68487/2711317	Diet and breast cancer.	No quality assessment.	The consumption of dietary fat, is probably suggestive of an increase in breast cancer risk, while studies evaluating the role of fruit/vegetable, meat as well as dietary patterns and	Diet seems to be modestly associated with the disease.	Further research should be oriented towards the analysis of nutrition patterns,	Different dietary assessments used in the studies.	5/7

**China 6**<br>**USA 3**<br>**Japan 3**<br>**Germany 3**<br>**Korea 2**<br>**Great Britain 2**<br>**France 1**<br>**Sweden 1**<br>**Japan/Brazil 1**<br><br>Missing land on the other studies		Range:<br>151/28380-11726/1280296<br><br>5 Case- control:<br>Total: 4875/5701<br>Range:<br>435/975-1350/1728<br><br>**11 Fruits & vegetables**<br>3 Cohort:<br>5999/370032<br>Range:<br>1072/32578-3659/285526<br><br>8 Case-control:<br>Total: 9901/10111<br>Range: 94/89-2503/3539<br><br>**14 Meat intake**<br>8 Cohort:<br>Total: 22402/780549<br>Range: 430/11699-7119-319826<br><br>6 Case-control:<br>Total: 8153/33835<br>Range:<br>94/89-3508/24697<br><br>**22 Soy product and isoflavones**<br>7 Cohort:<br>3681/254617<br>Range:<br>179/15555-1014/73223<br><br>15 Case control:<br>Total: 9439/66173<br>Range:<br>144/183-1070/36458<br><br>**9 Dietary fiber**<br>9 Cohort<br>Total:<br>15880/641322<br>Range:<br>324/11726-5461/185598<br><br>**5 Dietary carbohydrate**<br>3 Cohort:			breast cancer risk, provide inconsistent results.	No consistent and statistically strong association between breast cancer incidents and dietary factors, except for alcohol.	rather than specific foods.<br><br>Larger well-designed studies.	Difficult to make comparisons between studies.	

		Total:<br>3857/145301<br>Range: 289/8926-2952/74942<br><br>2 Case-control:<br>Total: 950/2782<br>Range:<br>475/475-1391/1391<br><br>**16 Dietary lipids**<br>8 Cohort:<br>Total: 17027/878396Range:<br>129/11726-7119/319826<br><br>8 Case-control:<br>Total: 8140/72704<br>Range:<br>56/224-3474/62573<br><br>This study has included more articles, but these are not shown in the tables.							
**Albuquerque et al., 2014**<br><br>Systematic review<br><br>**USA 6**<br>**Uruguay 3**<br>**China 2**<br>**France 2**<br>**Italy 2**<br>**Sweden 1**<br>**Canada 1**<br>**Japan 1**<br>**Brazil 1**<br>**Singapore 1**<br>**Korea 1**<br>**Australia 1**<br>**Germany 1**<br>**Cyprus 1**<br>**Tanzania 1**<br>**Three European countries 1**	To collate research on the topic of dietary patterns and breast cancer risks.	2001-2012<br><br>26 studies<br><br>11 Cohort:<br>Total: 15567/531747<br>Range: 207/1598-3026/90638<br><br>15 Case-control:<br>Total:<br>13395/39847<br>Range: 35/94-2884/22333	Breast cancer and dietary patterns risks.	PRISMA.<br><br>STROBE.	The findings of these studies suggest that Mediterranean dietary pattern and diets composed largely of vegetables, fruit, fish, and soy are associated with a decreased risk of breast cancer. Only one study showed a significant increase in risk associated with the Western dietary pattern.	There was no evidence of an association between traditional dietary patterns and risk of breast cancer. Diets that include alcoholic beverages may be associated with increased risk.	New studies are required.<br><br>Improvement of instruments.	The instruments used to collect data differ among the studies.<br><br>The questionnaire used in most cases is known to be subject to measurement error.<br><br>Few of the instruments were valid.	7/7
**Li et al., 2016**<br><br>Systematic review and Meta-analysis<br><br>**USA 4**<br>**Canada 1**	To test the hypothesis that dietary cholesterol intake increases the risk of breast cancer.	1987-2014<br><br>9 studies<br><br>6 Cohort:<br>Total: 5442/377909<br>Range: 54/3988-2830/182671	Dietary cholesterol intake and risk of breast cancer.	NOS.	The pooled relative risk with 95% confidence intervals of breast cancer for the highest versus lowest category of dietary cholesterol intake was 1.29 (1.06-1.56). For dose-response analysis, a nonlinear relationship was found between dietary cholesterol and breast	Results from this meta-analysis indicated that dietary cholesterol was associated with an increased risk of breast cancer.	In future investigations, 27HC, as a metabolite of cholesterol, should be paid more attention,	Recall bias.<br><br>Confounding factors.	7/7

**Finland 1**<br>**Uruguay 1**<br>**Saudi Arabia 1**		3 Case-control:<br>Total:<br>3226/9160<br>Range:<br>365/762-2362/7401			cancer, and the association became statistically significant when the cholesterol intake was greater than 370 mg/d.		especially in post-menopausal women with estrogen receptor-positive breast cancer.		
**Cao et al., 2016**<br><br>Systematic review and Meta-analysis<br><br>**Japan 2**<br>**Europe 1**<br>**USA 11**<br>**China 1**<br>**France 1**<br>**Sweden 3**<br>**Netherland 1**<br>**Italy 1**<br>**Norway 1**<br>**Canada 1**<br>**Finland 1**	To assess the association between dietary total fat and fatty acids intake and breast cancer risk	1987-2015<br><br>**Dietary intake:**<br>24 Cohort studies:<br>Total: 44629/1556599<br>Range:<br>54/270-10062/337237<br><br>**Biomarkers**:<br>7 Cohort studies:<br>Total: 1334/3511<br>Range: 71/212-363/1065	Dietary total fat and fatty acids/serum fat.	NOS.	The pooled RR of breast cancer for the highest versus lowest category of dietary total fat intake was 1.10 (1.02-1.19); however, no association was observed in studies adjusting for traditional risk factors of breast cancer. No association was observed between animal fat, vegetable fat, saturated fatty acids (SFA), monounsaturated fatty acids (MUFA), polyunsaturated fatty acids (PUFA), n-3 PUFA, n-6 PUFA, eicosapentaenoic acid, docosahexaenoic acid, alpha-linolenic acid, oleic acid, linoleic acid, arachidonic acid, and the risk of breast cancer. The pooled RRs of breast cancer for the highest versus lowest category of serum SFA, MUFA, PUFA, n-3 PUFA and n-6 PUFA were 1.00 (0.78-1.28), 1.41 (0.99-2.03), 0.59 (0.27-1.30), 0.81 (0.60-1.10) and 0.84 (0.60-1.18), respectively.	The meta-analysis suggest that dietary total fat and fatty acids might not be a major concern for breast cancer.	Studies with serum fatty acids are still needed to confirm the findings.	Confounders.<br><br>Limited number of studies with serum fatty acids.<br><br>Measurement errors.	7/7
**Kolahdouz Mohammadi et al., 2017**<br><br>Systematic review and Meta-analysis<br><br>**Finland 3**<br>**USA 1**<br>**Sweden 1**	To conduct a systematic review and meta-analysis assessing the association of c9,t11-CLA, and t11-18:1 VA (intake or serum levels) with breast cancer risk.	2000-2009<br><br>5 studies<br><br>2 Cohort<br>Total: 3893/129224<br>Range: 941/62573-2952/66651<br><br>3 Case-control:<br>Total: 1444/2486<br>Range: 127/204-1122/2036	Ruminant trans-fatty acids and risk of breast cancer.	NOS.	Three studies on c9,t11-CLA and t11-18:1 VA serum levels and t11-18:1 VA intake were evaluated in the systematic review only (narrative synthesis) and four studies (2 case-control and 2 cohort studies on c9,t11-CLA intake) were included in the meta-analysis (quantitative synthesis). The pooled RR for the highest versus lowest category of c9,t11-CLA intake was 0.94 (95% CI: 0.64-1.25) with evidence of heterogeneity (with 67,533 participants, I^2=78.3%, P=0.003). Studies that could not be included in the quantitative syntheses were inconclusive.	No association was found between c9,t11-CLA intake and breast cancer risk, but the number of studies identified was small.	Adequately powered observational studies specifically designed to assess the effects of R-TFA on breast cancer, are warranted.	Confounding or bias.<br><br>The meta-analysis, lacked power to perform subgroup and dose-response analysis.<br><br>In any meta-analysis, publication bias is possible.	7/7
**Yu et al., 2017**<br><br>Systematic review and Meta-analysis	To evaluate the association between dietary vitamin B2 intake and the risk of breast cancer.	1996-2015<br><br>10 studies	Dietary B 2 intake and breast cancer risk.	NOS.	No association between B2 intake and breast cancer risk.	Results indicate that dietary B2 intake is weakly related to reduced risk of breast cancer	More studies are still needed to further clarify B2 intake and breast cancer risk.	Confounding factors.<br><br>Assessment method.	7/7

**USA 3**<br>**Italy 2 Switzerland 1 Japan 1**<br>**Canada 1**<br>**China 1**<br>**Australia 1**		5 Cohort: Total: 5279/187303<br>Range: 391/9009-2491/72861<br><br>5 Case-control:<br>Total: 6989/7372<br>Range: 289/388-2569/2588<br><br>Enrollment period: 1982-2008						Recall bias in case control studies.	
**Wu et al., 2016**<br><br>Meta-analysis<br><br>**USA 15**<br>**Norway 5**<br>**Finland 1**<br>**Japan 6**<br>**Singapore 1**<br>**Denmark 2**<br>**Sweden 3**<br>**UK 2**<br>**China 2**<br>**Korea 1**<br>**France 3**<br>**Netherland 2**<br>**North American and Western Europe 1**<br>**European 2**	To investigate the association between different dietary protein sources and breast cancer risk.	1989-2015<br><br>46 studies<br><br>42 Cohort:<br>Total: 59362/2753808<br>Range: 29/1448-7379/500000<br><br>4 Case-control:<br>Total: 1253/27189<br>Range: 180/551-466/24697	Dietary protein.	NOS.	The summary relative risk (RR) for highest versus lowest intake was 1.07 (95% confidence interval (CI) 1.01-1.14, I(2) = 34.6%) for processed meat, 0.92 (95% CI 0.84-1.00, I(2) = 0%) for soy food, 0.93 (95% CI 0.85-1.00, I(2) = 40.1%) for skim milk, and 0.90 (95% CI 0.82-1.00, I(2) = 0%) for yogurt. Similar conclusions were obtained in dose-response association for each serving increase: total red meat (RR: 1.07; 95% CI 1.01-1.14, I(2) = 7.1%), fresh red meat (RR: 1.13; 95% CI 1.01-1.26, I(2) = 56.4%), processed meat (RR: 1.09; 95% CI 1.02-1.17, I(2) = 11.8%), soy food (RR: 0.91; 95% CI 0.84-1.00, I(2) = 0%), and skim milk (RR: 0.96; 95% CI 0.92-1.00, I(2) = 11.9%).	Higher total red meat, fresh red meat, and processed meat intake may be risk factors for breast cancer, whereas higher soy food and skim milk intake may reduce the risk of breast cancer. There was a null association between poultry, fish, egg, nuts, total milk, and whole milk intake and breast cancer risk.	Additional well-designed cohort or intervention studies and studies exploring the mechanisms are needed.	Most studies assumed that diet did not change over many years of follow up.	7/7
**Xiao et al., 2019**<br><br>Systematic review and Meta-analysis<br><br>**Greece 2**<br>**Spain 1**<br>**Argentina 1**<br>**Iran 1**<br>**France 2**<br>**Germany 1**<br>**China 2**<br>**Uruguay 3**<br>**Korea 1**<br>**USA 8**<br>**Italy 2**<br>**Japan 3**<br>**Canada 2**<br>**Australia 1**<br>**Sweden 1**	To examine the associations between different dietary patterns and the risk of breast cancer by conducting a meta-analysis of observational studies.	2001-2016<br><br>32 studies<br><br>14 Cohort:<br>Total: 26309/785507<br>Range: 119/1598-3659/91779<br><br>18 Case-control:<br>Total: 17344/44274<br>Range: 100/174-2884/22333	Dietary pattern and risk of breast cancer.	NOS.	The pooled analyses found that a Western dietary pattern was associated with a 14% increased risk (RR 1.14, 95% CI 1.02, 1.28), whereas a prudent dietary pattern was associated with an 18% reduced risk of breast cancer (RR 0.82, 95% CI 0.75, 0.89). In addition, sub-group analyses showed that the positive association between a Western dietary pattern and breast cancer risk was significant among post-menopausal (RR 1.20, 95% CI 1.06, 1.35), but not pre-menopausal women (RR 1.18, 95% CI 0.99, 1.40), and significant for hormone receptor-positive tumors (RR 1.18, 95% CI 1.04, 1.33), but not receptor-negative tumors (RR 0.97, 95% CI 0.83, 1.12). In contrast, the inverse association between a prudent dietary	The results of the current meta-analysis suggest a possible increased risk of breast cancer associated with a Western dietary pattern and a reduced risk with a prudent dietary pattern. As dietary patterns are modifiable, these findings may provide viable strategies for breast cancer prevention through changes in dietary intake.	Large-scale cohort studies of high-quality need to be conducted to further confirm the findings of the current meta-analysis.	Recall bias and control selection bias.<br><br>Confounding factors.<br><br>Different classification of the dietary patterns.<br><br>Variability in the factor analysis and/or principal component analysis may still exist.	5/7

**Three European counties: 1**					pattern and breast cancer was significant in pre-menopausal (RR 0.77, 95% CI 0.61, 0.98), but not post-menopausal women (RR 0.88, 95% CI 0.74, 1.03), and significant for both hormone receptor-positive and receptor-negative tumors.			
**Zahedi et al., 2018**<br><br>Systematic review and Meta-analysis<br><br>**Sweden 1**<br>**France 1**<br>**USA 3**<br>**Germany 1**<br>**Italy 2**<br>**China 1**	To summarize the findings on the association between dietary inflammatory potential (DIP) and the risk of breast cancer.	2015-2017<br><br>9 studies<br><br>6 Cohort:<br>Total: 16765/280855<br>Range: 145/1453-7495/122788<br><br>3 Case-control:<br>Total: 6323/15247<br>Range: 867/1691-2887/8399<br><br>Difficult to combine the numbers, because of classification.	Dietary inflammatory potential and breast cancer risk.	NOS.	The random effects meta-analysis showed a positive and significant association between DIP and the risk of breast cancer (pooled odds ratio, 1.14; 95% confidence interval, 1.01e1.27). The pooled effect size was not statistically significant because of the type of studies, including cohort (pooled relative risk, 1.04; 95% confidence interval, 0.98e1.10) and case-control (pooled odds ratio, 1.63; 95% confidence interval, 0.89e2.37) studies. We found a significant and positive association between higher DIP score and risk of breast cancer.	In conclusion, in this systematic review and meta-analysis of studies investigating the association between DIP and breast cancer, we found that DIP was positively and significantly associated with a risk of breast cancer.	No recommendations for further research.	Small number of studies.<br><br>Missing subgroup analysis.<br><br>Cofounding factors.	6/7
**Mulholland et al., 2008**<br><br>Systematic review and Meta-analysis<br><br>**USA 6**<br>**France 1**<br>**Italy 2**<br>**Australia 1**<br>**Denmark 1**<br>**Canada 1**<br>**Mexico 1**<br>**Switzerland 1**	To examine if an association exists between dietary glycaemic index (GI) and glycaemic load (GL) intake and breast cancer risk.	2001-2008<br><br>14 Studies<br><br>10 Cohort:<br>Total:<br>13122/485393<br>Range:<br>289/8959-4082/90655<br><br>4 Case-control:<br>Total:<br>4541/6618<br>Range:<br>331/534-2569/2588	Glycaemic load intake and breast cancer.	NOS.	Non-significant increased breast cancer risks for pre-menopausal women (relative risk (RR) 1.14, 95% CI 0.95-1.38) and post-menopausal women (RR 1.11, 95% CI 0.99-1.25) consuming the highest versus the lowest category of GI intake. Pooled cohort study results indicated no association between post-menopausal risk and GL intake (RR 1.03, 95% CI 0.94-1.12).	Findings do not provide strong support of an association between dietary GI and GL and breast cancer risk.	No recommendations for further research.	Confounders.<br><br>Each study in the meta-analysis had categorized GI and GL differently.	7/7
**Schlesinger et al., 2017**<br><br>Systematic review and Dose response Meta-analysis<br><br>**USA 10**<br>**France 1**<br>**China 1**<br>**Italy 2**<br>**Denmark 1**<br>**Sweden 1**	To investigate the associations between carbohydrate intake, glycemic index, glycemic load, and risk of breast cancer stratified by menopausal status, hormone receptor status, and body mass index (BMI).	1990-2015<br><br>19 studies<br><br>18 Cohort:<br>Total: 45752/1488402<br>Range: 15/590-11576/334849<br><br>1 Case- control: 56/214	Glycemic index, carbohydrate, BMI, and breast cancer risk.	No quality assessment.	The summary RRs (95% CIs) for breast cancer were 1.04 (1.00-1.07) per 10 units/d for glycemic index, 1.01 (0.98-1.04) per 50 units/d for glycemic load, and 1.00 (0.96-1.05) per 50 g/d for carbohydrate intake. For glycemic index, the association appeared slightly stronger among post-menopausal women (summary RR per 10 units/d, 1.06; 95% CI, 1.02-1.10) than among pre-menopausal women,	Menopausal and hormone receptor status, but not BMI, might be potential influencing factors for the associations between carbohydrate intake, glycemic index, glycemic load, and breast cancer risk.	Further studies on glycemic index, glycemic load, carbohydrate and sugar intake, and risk of breast cancer are needed. Such studies should account for	Cofounding factors.<br><br>There were no differences in associations detected between normal- and overweight pre- or post-menopausal women.	6/7

**European countries 1**<br>**Finland 1**<br>**Canada 1**					though the difference was not statistically significant (Pheterogeneity = 0.15). Glycemic load and carbohydrate intake were positively associated with breast cancer among post-menopausal women with estrogen-negative tumors (summary RR for glycemic load, 1.28; 95% CI, 1.08-1.52; and summary RR for carbohydrates, 1.13; 95% CI, 1.02-1.25). No differences in BMI were detected.		menopausal status, hormone receptor status, excess body weight, and use of hormone replacement therapy.	Measurement error of diet	
**Van Maele-Fabry et al., 2016**<br><br>Systematic review and Meta-analysis<br><br>**USA 2**<br>**Japan 2**<br>**Denmark 1**<br>**Sweden 1**	To investigate the association between dietary exposure to Cadmium (Cd) and breast cancer focusing on post-menopausal women	2012-2014<br><br>6 studies<br><br>5 Cohort:<br>Total: 11588/351617<br>Range:<br>402/23815-6658/150889<br><br>1 Case-control:<br>212/253<br><br>Enrollment period:<br>1987- 2002	Cadmium exposure and breast cancer.	No quality assessment.	No statistically significant increased risk of breast cancer was observed when all studies were combined (mRR. =. 1.03; 95% confidence interval [CI]: 0.89-1.19).	The study does not provide support for the hypothesis that dietary exposure to Cd increases the risk of breast cancer in post-menopausal women.	Further investigation with a focus on improved accuracy of individual dietary Cd intakes and differentiation of breast cancer by pathological features are needed.	Confounding variables.<br><br>Different dietary habits, quality of Cd data, Cd pollution levels, which could have impacted these studies differently.	6/7
**Wu et al., 2015**<br><br>Meta-analysis<br><br>**USA 2**<br>**Japan 2**<br>**Denmark 1**<br>**Sweden 1**	To perform an updated meta-analysis reevaluating the association between dietary Cadmium exposure and breast cancer risk.	2012-2014<br><br>6 Studies<br><br>5 Cohort:<br>Total: 11588/309585<br>Range: 402/23815-6658/150889<br><br>1 Case-control:<br>Total: 390/390	Cadmium exposure and breast cancer risk.	MOOSE.<br><br>STROBE.	There was no statistically significant positive association between dietary Cd exposure and BC risk, the combined RR and corresponding 95% CI was 1.01 [0.88, 1.14]. The result was not modified by menopause status, geographic area, or study design.	Our study did not find a statistically significant positive association between dietary Cd exposure and BC risk.	It is necessary to investigate this relationship among the high-risk groups, and more cohort studies based on diverse populations are needed.	Confounders.<br><br>Recall bias.<br><br>Measurement error.<br><br>Selection bias and publication bias.	7/7
**Lin et al., 2016**<br><br>Meta-Analysis<br><br>**USA 4**<br>**Sweden 1**<br>**Japan 3**<br>**Denmark 1**	To quantitatively summarize the current evidence for the relationship between Cadmium exposure and breast cancer risk.	2006-2014<br><br>10 studies<br><br>5 Cohort: Total: 11588/309585<br>Range:<br>402/23815-6658/150889<br><br>5 Case-control:<br>988/4294<br>Range:<br>99/99-390/3120	Cadmium exposure and breast cancer risk.	NOS.	Higher urinary Cadmium levels were associated with an increased risk for breast cancer (highest versus lowest quantile, pooled odds ratio [OR]=2.24, 95% confidence interval [95% CI]=1.49-3.35) and a 1mug/g creatinine increase in urinary cadmium led to a 1.02-fold increment of breast cancer (pooled OR=2.02, 95% CI=1.34-3.03); however, pooled estimates for dietary cadmium intake found no significant association between cadmium exposure and breast cancer risk (highest versus	The results suggest that cadmium exposure may lead to an increased risk of breast cancer, and urinary cadmium levels can serve as a reliable biomarker for long-term cadmium exposure and may predict the breast cancer risk.	Better designed population studies are needed. The underlying mechanisms for cadmium in promoting breast cancer development need more investigation and the corresponding	Measurement difficulties.<br><br>Recall bias.<br><br>Confounders.	7/7

					lowest quantile, pooled relative risk [RR]=1.01, 95% CI=0.89-1.15).		intervention methods should also be developed to prevent cadmium-induced breast cancer.		
**Larsson et al., 2015**<br><br>Systematic review and Meta-analysis<br><br>**United States 5**<br>**Japan 1**<br>**China 1**<br>**Lithuania 1**	To identify the association between urinary Cadmium concentration, a biomarker of Cadmium exposure, and breast cancer risk.	2006-2015<br><br>7 Studies<br><br>2 Cohort:<br>Total: 67/10 472<br>Range: 25/2254-42/8218<br><br>6 Case-Control:<br>Total: 1416/ 5 083<br>Range from 92/98-585/2884	Cadmium concentration and breast cancer risk.	No quality assessment.	There was no consistent association between urinary Cadmium and breast cancer mortality in the cohort studies. In case-control and cross-sectional studies, the pooled odds ratios were 2.24 (95% confidence interval: 1.50, 3.34; I^2 = 63.4%) for the highest versus lowest category of cadmium concentration and 1.66 (95% confidence interval: 1.23, 2.25) for each 0.5-mug/g creatinine increase of Cadmium concentration.	This meta-analysis suggests that a high cadmium exposure may be a risk factor for breast cancer.	Large prospective studies are needed to confirm this finding.	Most studies had a retrospective and cross-sectional design.<br><br>Selection bias.<br><br>Exposure measurement error.<br><br>Confounders.<br><br>Cadmium exposure differed between the countries.<br><br>Small studies.	6/7
**Hou et al., 2019**<br><br>Systematic review and Meta-analysis<br><br>**Sweden 5**<br>**USA 9**<br>**Australia 1**<br>**Europe 2**<br>**Germany 1**<br>**France 1**<br>**Singapore 1**<br>**UK 1**<br>**Japan 2**<br>**Netherlands 2**<br>**Canada 2**<br>**Italy 3**<br>**USA & Canada 1**	To estimate the pooled results of the association of healthy dietary patterns with breast cancer risk and survival.	2001-2016<br><br>31 Cohort studies:<br>Total:<br>56451/1574557<br>Range:<br>119/1598-10225/335062	Heathy dietary patterns	NOS.	There was a statistically significant lower risk of breast cancer associated with healthy dietary patterns (RR = 0.93, 95% CI: 0.88, 0.98). Subgroup analysis results suggested that there was an inverse association between breast cancer risk and posterior-derived healthy patterns, but no statistically significant associations were found in other stratified subgroups (a priori-derived diet, study region, menopausal status, or breast cancer subtypes). Healthy dietary patterns were associated inversely with all-cause mortality (RR = 0.76, 95% CI: 0.63, 0.92); however, no association was found for breast cancer specific mortality.	The results suggested that healthy dietary patterns might be associated with a reduced risk of breast cancer and all-cause mortality among breast cancer patients.	Further investigation is needed to better understand the mechanism between dietary patterns and breast cancer and how dietary patterns affect people differently by menopausal status and breast cancer subtypes. Future studies involving large scale randomized controlled trials or carefully designed observational studies are required to get	The included studies have their own strengths and limitations.<br><br>Only compared the risk estimates or mortality between the highest and lowest categories of healthy dietary patterns.<br><br>Only single time-point measurements of dietary patterns were examined in the included studies.<br><br>Only included published studies in English.	7/7

							more definitive conclusions.		
**Chang V.C. et al., 2019**<br><br>Systematic review and Meta-analysis<br><br>**USA 12**<br>**Canada 1**<br>**Germany 2**<br>**Denmark 1**<br>**Italy 1**<br>**UK 1**<br>**Switzerland 1**<br>**France 1**<br>**Finland 1**<br>**Sweden 1**<br>**China 2**<br>**Taiwan 1**<br>**Japan 1**<br>**Australia 1**	To evaluate the associations between both iron intake and body iron status and breast cancer risk.	1990-2018<br><br>27 studies<br><br>12 Cohort:<br>Total:<br>25870/867824<br>Range: 80/1795-9305/193742<br><br>15 Case-control: 13335/16816<br>Range:<br>107/212-3452/3474<br><br>Enrollment: 1969-2011	Iron intake, body iron status.	NOS.	Comparing the highest versus lowest category, heme iron intake was significantly associated with increased breast cancer risk, with a pooled RR of 1.12 (95% CI: 1.04–1.22), whereas no associations were found for dietary (1.01, 95% CI: 0.89–1.15), supplemental (1.02, 95% CI: 0.91–1.13), or total (0.97, 95% CI: 0.82–1.14) iron intake. Associations of iron status indicators with breast cancer risk were generally in the positive direction; however, a significant pooled RR was found only for serum/plasma levels (highest vs. lowest) of iron (1.22, 95% CI: 1.01–1.47), but not for ferritin (1.13, 95% CI: 0.78–1.62), transferrin saturation (1.16, 95% CI: 0.91–1.47), or total iron-binding capacity (1.10, 95% CI: 0.97–1.25). In addition, a nonlinear dose-response was observed for heme iron intake and serum iron (both Pnonlinearity < 0.05).	Heme iron intake and serum iron levels may be positively associated with breast cancer risk. Although associations were modest, these findings may have public health implications given the widespread consumption of (heme) iron-rich foods.	To better elucidate the relationship between iron and breast cancer risk. A larger number of homogeneous studies across a wide range of exposure values are needed to confirm these results in the future	The meta-analysis for some iron measures was based only on a small number of studies.<br><br>Restricted cubic spline dose-response analyses were limited to the range of exposure values derived from individual studies.<br><br>Analyses combined risk estimates across studies with different designs, populations, settings, statistical adjustments of covariates, etc. (some of which were explored in our subgroup analyses).<br><br>Genetic association studies were not considered.	7/7

Table S6. Blood and Metabolism.

**Author, year, country Methodology**	**Aim**	**Period of included studies**<br><br>**Number of included studies**<br><br>**Number of persons included in the studies**	**Theme/subjects of the different studies**	**Quality assessment**	**Results Risks and causes**	**Conclusions**	**Further research**	**Limitations**	**CASP scores**
**Macis et al., 2014**<br><br>Systematic review and Meta-analysis<br><br>**USA 3**<br>**Japan 1**<br>**Greece 2**	To investigate whether circulating adiponectin, an insulin-sensitizing hormone produced by adipocytes, is associated with breast cancer risk or not.	2003-2013<br><br>15 studies<br><br>1 Cohort:<br>Total:<br>57/235	Circulating adiponectin.	MOOSE.<br><br>STROBE.	The SRR for the 'highest' versus 'lowest' adiponectin levels indicated a 34% reduction in breast cancer risk [95% confidence interval (CI): 13%-50%]. Between-study heterogeneity was not substantial (I^2=53%). The comparison between 'highest' and 'lowest' levels of adiponectin	Low circulating adiponectin levels are associated with an increased breast cancer risk.	Properly designed studies are needed to confirm the role of adiponectin as breast cancer biomarker, and clinical trials	Confounders	7/7

**China 1**<br>**Korea 1**<br>**Germany 1**<br>**Sweden 1**<br>**Taiwan 1**<br>**Malaysia 1**<br>**Italy 1**<br>**France 1**<br>**Saudi Arabia 1**		14 Case-control:<br>Total: 4192/5042<br>Range:<br>41/41-1477/2196			showed an inverse association in post-menopausal women (SRR=0.80; 95% CI: 0.63-1.01) and an indication of an inverse relationship in pre-menopausal women (SRR=0.72, 95% CI: 0.30-1.72).		should be performed to identify those interventions that may be effective in modulating adiponectin levels and reducing breast cancer risk.		
**Yu, Z. et al., 2019**<br><br>Meta-analysis<br><br>**Pakistan 1**<br>**Kuwait 2**<br>**Saudi Arabia 2**<br>**Taiwan 1**<br>**Portugal 1**<br>**Sweden 1**<br>**Greece 3**<br>**USA 3**<br>**Turkey 3**<br>**UK 1**<br>**China 3**<br>**German**<br>**Japan 2**<br>**Mexico 1**<br>**Malaysia 1**<br>**France 1**	To assess the association of adiponectin with breast cancer.	2003-2017<br><br>27 Case-control studies:<br>Total:<br>7 176/ 8 318<br>Range:<br>Case-control:<br>40/30-1447/2196	Adiponectin and risk of breast cancer.	NOS.	Overall, there was an evident inverse association between serum adiponectin levels and breast cancer (MD=-0.29, 95% CI=(-0.38, -0.21), P<.001). Asian subgroup showed a significant negative association between serum adiponectin concentrations and breast cancer in subgroup analysis by ethnicity (MD=-2.19, 95% CI=(-3.45, -0.94), P<.001). However, no statistical significance was found in Caucasian subgroup (MD=-0.65, 95% CI=(-1.47, 0.17), P=0.12). Additionally, a further subgroup analysis of Asian stratified by menopausal status showed higher concentrations of adiponectin in healthy control group, whether they were pre-menopausal (MD=-0.85, 95% CI=(-1.50, -0.19), P=.01) or post-menopausal (MD=-2.17, 95% CI=(-4.17, -0.18), P=.03). No significant difference was observed concerning the association between serum adiponectin and breast cancer metastasis (MD=-1.56, 95% CI=(-4.90, 1.78), P=.36).	The current meta-analysis suggests that the serum adiponectin may be inversely associated with breast cancer. Decreased serum adiponectin levels in pre-menopausal women may also be inversely associated with breast cancer risk other than post-menopausal status. In addition, low serum adiponectin levels in Asian women were more likely to be associated with breast cancer risk than Caucasian women.	A further meta-analysis might be essential in the future, because of the limitations. A larger sample based meta-analysis is essential to be carried out in the future for further investigation.	Only included observational studies.<br><br>Some studies might be missed.<br><br>Failed to carry out further subgroup analyses.<br><br>Confounding factors.	7/7
**Ye J. et al., 2014**<br><br>Systematic review and Meta-analysis<br><br>**China 1**<br>**Taiwan 2**<br>**Malaysia 1**<br>**Korea 1**<br>**Japan 1**<br>**Greece 2**	The aim of the present study was to conduct a systematic review and a meta-analysis on the association between circulating adiponectin levels and the risk of breast cancer.	2003-2010<br><br>8 Case-control studies:<br>Total: 885/918<br>Range: 41/43-244/244	Circulating adiponectin and breast cancer.	MOOSE (but did not report).	Serum total adiponectin concentrations were lower in patients with breast cancer, with a pooled SMD of -0.39 mug/ml (95% CI -0.618 to -0.161, P=0.001). However, adiponectin levels were not associated with the risk of breast cancer in pre-menopausal women [four studies, random effects SMD=0.02 mug/ml (95% CI -0.164 to 0.204, P=0.829)].	These results collectively suggest that lower adiponectin levels are associated with a higher risk of breast cancer in post-menopausal women.	More research is needed to clarify the association between visceral fat and circulating adiponectin levels.	Analyses based on observational studies.<br><br>Confounding factors.<br><br>Recall and selection bias.	6/7

								Small studies with null results would not be published.	
**Gu, L. et al., 2018**<br><br>Meta-analysis<br><br>**Turkey 2**<br>**Greece 2**<br>**Portugal 1**<br>**Japan 2**<br>**China 5**<br>**USA 6**<br>**Saudi Arabia 2**<br>**Pakistan 1**<br>**France 1**<br>**Mexico 1**<br>**Kuwait 2**<br>**Malaysia 1**<br>**Germany 2**<br>**Australia 1**<br>**Korea 1**<br>**Taiwan 1**	Updated meta-analysis was conducted to assess the association between serum adiponectin concentration and BC risk by precise results.	2003-2017<br><br>31 Case-control studies: Total: 7 388/8 491 Range: 40/30-1167/1575	Serum adiponectin concentration and breast cancer risk	NOS.	The overall results indicated that serum adiponectin levels in BC cases were significantly lower than the controls (SMD=0.33, P<0.0001). As for the subgroup analysis of menstrual status, serum adiponectin levels were significantly lower in pre- and post-menopausal BC cases. Moreover, the subgroup analysis by ethnicity in pre- and post-menopausal group indicated an inverse association between adiponectin levels and BC risk in Asian population, but not in Caucasian population.	The present meta-analysis suggests that low serum adiponectin concentration may be associated with an increased BC risk in pre-menopausal and post-menopausal women, especially among Asians. Adiponectin may serve as a biomarker of BC risk and help to identify subjects at high risk for BC development.	Further investigation is needed to explore a threshold of adiponectin which could can have a protective effect against BC. Moreover, adiponectin may serve as a biomarker of BC risk and help to identify subjects at high risk for BC development. More rigorous and uniform case–control is necessary to confirm these results.	Missing subgroup analysis.<br><br>Only observational studies, which may have not been completely controlled for confounders.	7/7
**Pan et al., 2018**<br><br>Systematic review and Meta-analysis<br><br>**Sri Lanka 1**<br>**China 3**<br>**Egypt 1**<br>**Greece 5**<br>**Portugal 1**<br>**Saudi Arabia 2**<br>**UK 1**<br>**Iran 1**<br>**Mexico 2**<br>**US 6**<br>**France 1**<br>**Germany**<br>**Turkey 3**<br>**Italy 1**<br>**Australia 1**<br>**China/Taiwan 2**<br>**Norway 1**<br>**Korea 1**	We conducted an updated meta-analysis to investigate the role of leptin in breast cancer.	2000-2017<br><br>34 Case-control studies: Total: 6 033/7 589 Range: 30/30-875/1024	Leptin and breast cancer risk.	MOOSE/NOS.	Serum leptin levels were related to breast cancer risk as demonstrated by calculations of the overall SMD=0.46 (95% CI=0.31-0.60, I^2=93.5%). A subgroup analysis of BMI identified an association between breast cancer and serum leptin levels in patients who are overweight and obese (overweight: SMD=0.35, 95% CI=0.13–0.57, I^2=88.1%; obesity: SMD=1.38, 95% CI=0.64–2.12, I^2=89.6%). Additionally, menopausal status subgroup analysis revealed a significant association in post-menopausal women (SMD=0.26, 95% CI=0.12–0.40, I^2=77.9%). Furthermore, we identified a significant association between breast cancer and serum leptin levels in Chinese women (SMD=0.61, 95% CI=0.44– 0.79, I^2=40.6%).	The results of this meta-analysis suggested that leptin could be a potential biomarker for breast cancer risk in women, especially overweight/obese or post-menopausal women. Therefore, it may be useful for identifying subjects with a high risk for breast cancer who may benefit from preventive treatments.	The power of this subgroup analysis was weak, and further research is required. Extensive sample size studies are needed to be conducted for eliminating the confounding factor of participation rates of controls.	No subgroup analyses.<br><br>Only studies published in English.<br><br>The inclusion rates of hospital and population groups in the studies were 94.29% (33 articles) and 5.71% (2 items), respectively.<br><br>The participation rate of controls is an essential factor for assessing selection bias.	7/7

**Shekarriz-Foumani & Khodaie, 2016**<br><br>Systematic review<br><br>**USA 4**<br>**Iran 3**<br>**Egypt 1**<br>**Thailand 1**<br>**Korea 1**<br>**Pakistan 1**<br>**China 1**<br>**Turkey 1**	To evaluate the correlation of plasma 25-hydroxyvitamin D deficiency with breast neoplasms risk among women.	2014-2015<br><br>13 studies<br><br>11 Case-control studies:<br>Total:<br>9015/20998<br>Ranges: 60/49-3634/17133<br><br>2 Cross-sectional<br>Total: 386<br>Range: 186-200	Vitamin D level.	NHLBI.	Definition of low vitamin D levels varied greatly among studies, making comparisons difficult, but most of them have defined deficiency as 25(OH)D < 20 ng/mL. Evidence was mainly of fair quality.	This study has provided evidence that vitamin D deficiency has been very prevalent in patients with breast neoplasms, more than comparable matched control population, and risk of breast cancer has increased with low vitamin D levels.	There is need for high quality studies that assess the health consequences attributable to vitamin D deficiency employing standard definitions.	Variation in quality variabilities in definitions of deficiency of variables.<br><br>Difficult to make comparisons.	7/7
**Yin, et al., 2010**<br><br>Systematic review and Meta-analysis<br><br>**USA 6**<br>**Germany 2**<br>**UK 1**<br>**Denmark 1**	To review and summarize observational epidemiological studies regarding the association between serum vitamin D (measured as 25(OH)D levels) and the risk of breast cancer.	2005-2009<br><br>10 studies<br><br>1 Cohort:<br>Total: 28/9185<br><br>9 Case-control:<br>Total: 6147/l 6754<br>Range: 242/179-1394/1365	Vitamin D level.	No quality assessment.	In meta-analyses, summary RRs (95% confidence interval (CI)) for an increase of 25(OH)D by 20 ng/ml were 0.59 (0.48-0.73), 0.92 (0.82-1.04) and 0.73 (0.60-0.88) with P values of <0.001, 0.164 and 0.001 for case-control studies, nested case-control studies and both study designs combined, respectively.	While case-control studies with measurement of 25(OH)D after diagnosis suggest an inverse association, a statistically significant inverse association remained unconfirmed in prospective studies with measurement of 25(OH)D years before diagnosis.	Further studies are needed to clarify the potential role and the relevant exposure time regarding vitamin D and breast cancer risk.	Heterogeneity between studies.<br><br>Individual-level data has not been available, different results reported.<br><br>Different studies measurements.<br><br>May have missed a relevant study in the search.	6/7
**Tommie et al., 2018**<br><br>Systematic review<br><br>**Germany 1**<br>**USA 7**<br>**Saudi Arabia 1**<br>**Korea 1**<br>**Denmark 1**<br>**New York and Sweden 1**<br>**Sweden 1**<br>**10 European countries 1**	To conduct a systematic review to answer the question, "Is there a relationship between lower serum/plasma vitamin D levels and increased risk of triple negative breast cancer (TNBC) specifically?"	2009-2016<br><br>14 Case-control studies:<br>Total:<br>13 135/28 326<br>Range: 142/194-1585/17133	Serum vitamin D and breast cancer risk.	No quality assessment.	Fourteen studies met our criteria, including case-control, nested case-control, and case-series studies, reflecting the cumulative results of 13,135 breast cancer cases. When grouped by relevancy to TNBC, the proportion of analyses across all study types showing a significant association between vitamin D status and breast cancer diagnosis was 37% for non-TNBC analyses, 48% for analyses that included some TNBC cases, and 88% for TNBC analyses.	Our results suggest that low vitamin D status may particularly increase the risk of TNBC, although more research is needed to determine if this association is causative. Women should be routinely screened for 25(OH)D deficiency.	More research is needed to determine if this association is causative.	Missing subgroup analysis.<br><br>Different measures in the studies.<br><br>Temporal associations between the duration of 25(OH)D deficiency and breast cancer risk cannot be determined.<br><br>Overrepresented of white/Caucasian race/ethnicity.	6/7
**Hu et al., 2015**<br><br>Meta-analysis	To summarize the associations between plasma retinol,	1987-2010<br><br>40 Case-control studies:	Plasma level of retinol(vit-A) and vitamins.	MOOSE.	No significant association between plasma retinol and breast cancer was observed (p = 0.13). Significant	Severe alpha-tocopherol deficiency could increase breast	No suggestions for future research.	Confounding factors in included studies.	7/7

**Kuwait 1**<br>**Iran 1**<br>**Saudi Arabia 1**<br>**Algerie 1**<br>**Canada 2**<br>**Australia 1**<br>**India 5**<br>**Argentina 1**<br>**Italy/France 1**<br>**France 3**<br>**Taiwan 1**<br>**Korea 3**<br>**USA 9**<br>**Italy 1**<br>**Netherlands 1**<br>**Turkey 2**<br>**Malaysia 1**<br>**China 1**<br>**Sweden 1**<br>**Finland 2**<br>**England 1**	vitamins A, C, and alpha-tocopherol, and breast cancer risk.	Total: 6561/13157<br>Range:<br>11/11-969/5260			association was observed between plasma alpha-tocopherol and breast cancer (pooled OR 0.42, 95% CI 0.25, 0.72, p = 0.00) in the subgroup with the median lowest level of 5.74-9.16 mumol/L. No significant association between plasma retinol and vitamin A and breast cancer was observed.	cancer risk. The association between plasma vitamin C and breast cancer was only significant in case-control studies. There was no significant association between other vitamins and breast cancer risk.		High heterogeneity in the analysis in the included studies.	
**Wulaningsih et al., 2016**<br><br>Systematic review and Meta-analysis<br><br>**USA 1**<br>**Sweden 1**	To investigate the association between serum Calcium and risk of breast cancer.	2007-2016<br><br>3 Cohort studies:<br>Total: 239859<br>Range: 2338-229674<br><br>Not reported case/control	Serum Calcium	Not reported.	We found an inverse association between total serum Calcium and breast cancer when comparing the fourth quartile to the first quartile (HR: 0.94, 95% CI: 0.88-0.99, p value for trend 0.04) and similar results using albumin-corrected Calcium.	Higher baseline serum Calcium was associated with a lower risk of a breast cancer diagnosis. There is an inverse association between serum calcium and breast cancer risk.	Further studies should include mechanistic investigations.	Confounders.<br><br>Lack of information on free ionized Calcium.<br><br>Only single measurement of Calcium.<br><br>Few studies, only from Sweden and USA.	6/7
**Esposito et al., 2013**<br><br>Systematic review and Meta-analysis<br><br>**USA 3**<br>**Italy 2**<br>**Japan 1**<br>**Uruguay 1**<br>**Italy/**<br>**Switzerland 1**<br>**Austria/**	To assess metabolic syndrome (MS) and its individual components in post-menopausal breast cancer (PBC).	2009-2012<br><br>9 studies<br><br>5 Cohort:<br>Total:<br>2010/366284<br>Range: 42/713-5450/287320<br><br>4 Case-control:<br>Total: 4407/5261<br>Range:163/261-3869/4082	Metabolic syndrome.	NOS.	MS was associated with a 52% increase in cancer risk (P < 0.001) - for the most part confined to noncohort studies (109% increased risk); the risk estimates changed little, depending on populations (United States and Europe) and definition of the syndrome (traditional vs non-traditional). The risk estimates for PBC were 1.12 (P = 0.068) for higher values of body mass index/waist circumference, 1.19 (P = 0.005) for hyperglycemia (higher fasting glucose or diabetes),	MS is associated with a moderately increased risk of PBC. No single component explains the risk conveyed by the full syndrome.	Further studies should adopt the same definition of MS and aim for a more comprehensive consideration of covariates to allow for a far more comprehensive control of confounding.	Confounding variables<br><br>Different design, components and cut off.<br><br>Possibility of publication bias.<br><br>Null results not published	7/7

**Sweden/ Norway 1**					1.13 (P = 0.027) for higher blood pressure, 1.08 (P = 0.248) for higher triglycerides, and 1.39 (P = 0.008) for lower high-density lipoprotein cholesterol.				
**Bhandari et al., 2014**<br><br>Systematic review and Meta-analysis<br><br>**Italy 3**<br>**USA 2**<br>**Japan 2**<br>**Uruguay 1**<br>**Italy/Switzerland 1**	To report a systematic review and meta-analysis of the association between metabolic syndrome (MS) and breast cancer risk in all adult females.	2008-2012<br><br>9 studies<br><br>5 Cohort:<br>Total: 1689/92021<br>Range:<br>77/4888-1228/49172<br><br>4 Case-control:<br>Total 4399/9655<br>Range: 163/792-1988/4093	Metabolic syndrome.	STROBE.	A modest, positive association was observed between MS and breast cancer risk (RR: 1.47, 95% CI, 1.15-1.87; z = 3.13; p = 0.002; Q = 26.28, p = 0.001; I 2 = 69.55%).	MS is associated with increased breast cancer risk in adult women.	Future research should comprise analysis based definition of MS and employ objective and standard biomarkers for assessing each MS component. Additionally, to control for the association of BC and adiposity as a possible confounder.	Different methods used to assess exposure, identify cancer, and control for cofounders.<br><br>Small number of studies.<br><br>Different study design, and varied population studied.	7/7
**Guo et al., 2015**<br><br>Meta-analysis<br><br>**USA 6**<br>**Netherland 1**<br>**England 1**<br>**Denmark 1**<br>**Sweden 1**<br>**China 2**<br>**France 2**<br>**KSA 1**	To conduct the associations between elevated C-reactive protein (CRP) and breast cancer risk.	2005-2014<br><br>15 studies<br><br>7 Cohort:<br>Total: 2781/379902<br>Range:<br>33/1241-2438/274703<br><br>8 Case-control:<br>Total: 2505/25055<br>Range: 56/109-709/19437	C-reactive protein.	No quality assessment.	There was moderate heterogeneity among studies (I(2)= 45.9%). The association was stronger in Asian population (OR = 1.57, 95% CI: 1.25-1.96) compared to European (OR = 1.12, 95% CI: 1.02-1.23) and American (OR = 1.08, 95% CI: 1.01-1.16). Prediagnostic high-sensitivity CRP concentrations (OR = 1.22, 95% CI: 1.10-1.35) was superior to common CRP (OR = 1.08, 95% CI: 1.01-1.15) in predicting breast cancer risk.	The meta-analysis indicated that elevated CRP levels was associated with increased risk of breast cancer.	Further research effort should be performed to identify whether CRP, as a marker of inflammation, plays a direct role in breast carcinogenesis.	Heterogeneous study included,<br><br>Relevant information is missing in included studies.<br><br>Confounding factors	6/7
**Chan et al., 2015**<br><br>Systematic review and Meta-analysis<br><br>**USA 6**<br>**France 2**<br>**China 1**<br>**UK 1**<br>**Netherlands 1**<br>**Denmark 1**	To explore the association between circulating C-reactive protein (CRP), a low-grade inflammation biomarker, and breast cancer risk.	2005-2015<br><br>12 Studies<br><br>7 Cohort:<br>Total: 1627/65103<br>Range: 33/1305-892/19437<br><br>5 Case-control:<br>Total: 1860/2647<br>Range: 85/163-706/1040<br><br>Enrollment period: 1989-2007	If CRP has a causal role in cancer or is a marker of disease.	PRISMA, but no quality assessment.	For each doubling of CRP concentration, a 7% [95% confidence interval (CI), 2%-12%] and 6% (95% CI, 1%-11%) increased risk was observed (I^2 = 47% and 32%; Heterogeneity = 0.04 and 0.17), respectively. On average, associations in studies adjusted for lifestyle factors were attenuated. The associations in studies adjusted or not adjusted for body mass index were similar.	Low-grade inflammation may have a role in breast cancer development.	Additional prospective studies are needed to better understand confounding and effect modification from lifestyle factors.	Pre-menopausal women are underrepresented.<br><br>The associations in studies that were unadjusted for HT use, physical activity, or alcohol use appeared stronger than adjusted.	6/7

**Touvir et al., 2015**<br><br>Systematic review and Meta-analysis<br><br>**USA 7**<br>**Sweden 2**<br>**Finland 1**<br>**Norway 3**<br>**Denmark**<br>**Iceland**<br>**Japan 3**<br>**Italy 1**<br>**Austria, Norway & Sweden 2**<br>**France 2**<br>**Korea 1**<br>**Europe 13**<br>**America 7**<br>**Asia 4**	To conduct systematic review and meta-analysis of prospective studies investigating the associations between total cholesterol (TC), HDL-cholesterol (HDL-C) and LDL-cholesterol (LDL-C) levels and the risk of breast cancer.	1986-2014<br><br>24 studies<br><br>22 Cohort: Total: 31 918/1 488 200 Range: 31/6105-3597/433115<br><br>2 Case-control: Total: 359/1 284 Range: 163/492-196/792<br><br>Enrollment period: 1963-2005	Cholesterol.	NOS.	The summary HR for the association between TC and breast cancer risk was 0.97 (95% CI 0.94, 1.00; dose-response per 1 mmol/l increment,). HDL-C and breast cancer risk was 0.86 (95% CI 0.69, 1.09; dose-response per 1 mmol/l increment, six studies), with high heterogeneity (I 2= 67 and 47%, respectively). For studies that eliminated preclinical bias, an inverse association was observed between the risk of breast cancer and TC (dose-response HR 0.94 (95% CI 0.89, 0.99), seven studies, I 2= 78%; highest v. lowest HR 0.82 (95% CI 0.66, 1.02), nine studies, I 2= 81%) and HDL-C (dose-response HR 0.81 (95% CI 0.65, 1.02), five studies, I 2= 30%; highest v. lowest HR 0.82 (95% CI 0.69, 0.98), five studies, I 2= 0%). There was no association observed between LDL-C and the risk of breast cancer (four studies)	The present meta-analysis confirms the evidence of a modest but statistically significant inverse association between TC and more specifically HDL-C and the risk of breast cancer, supported by mechanistic plausibility from experimental studies.	Further large prospective studies that adequately control for preclinical bias are needed to confirm the results on the role of cholesterol level and its fractions in the etiology of breast cancer.	Confounding, relatively few studies.<br><br>Lack of information about therapeutic strategies.<br><br>Only a single measurement of cholesterol.<br><br>Only search in Medline.	7/7
**Ni et al., 2015**<br><br>Meta-analysis<br><br>**Denmark 1**<br>**Norway 2**<br>**USA 4**<br>**Japan 2**<br>**Europa 2**<br>**France 2**<br>**Korea 1**<br>**Sweden 1**	To explore the causal associations between serum lipids and breast cancer risk.	1992-2012<br><br>15 Cohort studies: Total: 29361/ 1529363 Range: 51/4433-6105/433115	Serum lipids.	NOS MOOSE.	RRs of breast cancer for the highest versus lowest categories were 0.96 (95% CI: 0.86-1.07) for TC, 0.92 (95% CI: 0.73-1.16) for HDL-C, 0.90 (95% CI: 0.77-1.06) for LDL-C, and 0.93 (95% CI: 0.86-1.00) for TG. Notably, for HDL-C, a significant reduction of breast cancer risk was observed among post-menopausal women (RR = 0.77, 95% CI: 0.64-0.93) but not among pre-menopausal women. Similar trends of the associations were observed in the dose-response analysis.	Our findings suggest that serum levels of TG but not TC and LDL-C may be inversely associated with breast cancer risk. Serum HDL-C may also protect against breast carcinogenesis among post-menopausal women.	Future studies with larger sample sizes, detailed menopausal status information and consistent adjustments for confounders, especially among Asian populations is warranted.	Confounders.<br><br>The adjusted covariates differ for each of the studies.	7/7
**Miao et al., 2014**<br><br>Meta-analysis<br><br>**UK 2**<br>**USA 3**<br>**India 1**<br>**Norway 1**<br>**Iceland 1**<br>**China 3**	To investigate whether the risk of breast cancer is higher in any specific ABO blood type.	1954-2011<br><br>14 Case-control studies: Total: 9972/247 863 Range: 76/300-2548/79699	ABO blood type.	No quality assessment.	Relative to blood type O, women with blood type A (odds ratio (OR) = 1.115, 95% confidence interval (CI) 0.992-1.254), B (OR=0.983, 95% CI 0.915-1.056) and AB (OR=1.042, 95% CI 0.881-1.231) had the same breast cancer risk. The risk for women with Rhesus-positive (Rh+) was the same as those with Rh-negative (Rh-) (OR=0.948, 95% CI 0.667-1.348).	This meta-analysis suggests Caucasians with blood type A may have a higher risk of breast cancer than other Caucasians. No association was found in any other blood type or any other population. Similarly,	Nothing recommended.	The results are based on unadjusted OR.<br><br>There is a possibility of incomplete retrieval or abstraction of data.	6/7

**Uruguay 1**<br>**Turkey 1**<br>**Greece 1**					Among Caucasians, the OR of blood type A was 1.066 (95% CI, 1.001-1.134, P=0.522 for heterogeneity).	the Rh factor had no association with the risk of breast cancer.		There is both old and relatively new studies included in this study.	
**Lee et al., 2017**<br><br>Systematic review<br><br>**USA 7**<br>**Malaysia 1**<br>**China 3**<br>**Denmark 1**<br>**France 1**<br>**Finland 3**<br>**Spain 2**<br>**Poland 1**	To summarize the published literature on oxidative stress biomarkers and breast cancer.	2002-2015<br><br>19 studies<br><br>8 Cohort:<br>Total: 1023<br>Range: 30/363<br><br>11 Case- control:<br>Total:4801/6081<br>Range: 57/70-1050/1108<br><br>Enroll period: 1989-2006	Oxidative stress and biomarkers.	No quality assessment.	We identified eleven case-control studies of oxidative stress biomarkers and breast cancer. Biomarkers utilized varied, and menopausal status was a key modifying factor. Across three nested case-control studies with biomarkers measured before diagnosis, one reported increased risk of post-menopausal breast cancer in association with 8-oxodG (DNA damage biomarker), while two (one of F2-isoprostanes and one of fluorescent oxidation products) reported inverse associations for pre-menopausal breast cancer only. We identified eight prognostic studies. Two reported associations for lipid peroxidation and breast cancer prognosis, while results for other studies were null.	DNA damage may increase risk of breast cancer among post-menopausal women, while lipid peroxidation may be inversely associated with pre-menopausal breast cancer. Lipid peroxidation may be associated with survival after breast cancer diagnosis.	These results require evaluation in large, prospective cohort studies.	Lack of studies in minority populations.<br><br>No studies evaluated associations by breast cancer molecular subtype.<br><br>Most did not evaluate multiple valid biomarkers.<br><br>Insufficient data to make a meta-analysis at this time.	6/7
**Jouybari, L. et al., 2018**<br><br>Meta-analysis<br><br>**Finland 1**<br>**Czech/Germany 1**<br>**Nigeria 1**<br>**Poland 1**<br>**Turkey 1**<br>**India 2**<br>**Pakistan 2**<br>**Canada 1**<br>**Spain 1**<br>**USA 4**<br>**Portugal/Germany 1**	Studying the link between Arsenic (As), Cadmium (Cd), and Nickel (Ni) concentrations and brast cancer by using a meta-analysis.	1984-2015<br><br>16 Case-control studies:<br>Total: 1187/922. Range: 12/8-433/433	Toxic elements and breast cancer risk	Not reported.	The overall integration of data from the 3 groups led to the conclusion that there was a significant difference in Cd and Ni statuses between healthy and breast cancer patients; the standard mean difference was 2.65 (95% CI: 1.57–3.73; P=0.000) and 2.06 (95% CI: 1.20–3.32; P=0.000), respectively. Whereas, there was no significant statistical difference in As status between healthy subjects and breast cancer patients; the standard mean difference between them being 0.52 (95% CI: –0.12–1.16; P=0.114).	The present study indicates that there is a direct and positive association between Cd and Ni concentrations and BC risk. It is a warning to health care providers and policy makers to find viable solutions and take requisite measures to reduce BC risk in the society.	Nothing recommended	All methods of variance measurement were not uniform.<br><br>Not enough information was available about the lifestyle and nutrition of the participants.<br><br>Different kinds of screening methods	6/7
**He et al., 2015**<br><br>Meta-analysis<br><br>**USA 9**<br>**UK 3**<br>**Italy 2**<br>**Australia 1**	To evaluate the association between sex hormone binding globulin (SHBG) level and risk of breast cancer in post-menopausal women.	1996-2014<br><br>21 Case-control studies<br>Total: 5007/10267<br>Range from 24/88-677/1309<br><br>Enrollment period: 1968-2004.	Association between sex hormone binding globulin level and risk of breast cancer.	NOS.	In post-menopausal women, the pooled RR for breast cancer comparing highest with lowest categories of SHBG was 0.64 (95% CI 0.57-0.72, p<0.001, I(2)=6.5%). The pooled RRs were not obviously altered in the sensitivity analyses and subgroup analyses. In	The findings from this meta-analysis suggest that high SHBG level is significantly associated with decreased risk of breast cancer in post-menopausal women,	Further prospective studies are needed to identify the association between SHBG and risk of breast cancer in Asian	The lack of individual participant data.<br><br>Predominant white woman included in these studies.	7/7

**Denmark 1**<br>**Sweden 1**<br>**Different countries 3**<br>**Japan 1**					cumulative meta-analysis, a more significant association between SHBG level and risk of breast cancer in post-menopausal women was observed as evidence accumulated by publication year.	and it's a protective factor of breast cancer in post-menopausal women.	and African groups.		
**Estébanez, N., et al., 2018**<br><br>Systematic review and Meta-analysis<br><br>**Sweden 2**<br>**Canada 4**<br>**USA 25**<br>**France 4**<br>**Europe 3**<br>**Denmark 2**<br>**USA & Sweden 1**<br>**Germany 5**<br>**Iran 6**<br>**Australia 1**<br>**Norway 2**<br>**China 2**<br>**Mexico 1**<br>**UK 1**<br>**Brazil 1**<br>**Korea 1**<br>**India 2**<br>**Saudi Arabia 1**<br>**Japan 1**<br>**Taiwan 1**<br>**Switzerland 1**<br>**Italy 1**	Examines the effects of the 25(OH)D, 1,25(OH)2D, and vitamin D intake on breast cancer risk.	1998-2018<br><br>69 studies<br><br>**43 Serum vitamin D status studies:**<br>6 Cohort studies:<br>Total:<br>3502/24606<br>Range: 63/2471-1600/5606<br>37 Case-control studies: Total: 67348<br>Range: 200-20767<br><br>**26 Vitamin D intake studies:**<br><br>11 Cohort studies:<br>Total: 25733/ 804239<br><br>Range: 190/5009-7760/319985<br><br>15 Case-control studies:<br>Total: 355741<br>Range: 152-323612	Vitamin D.	No quality assessment.	Study showed a protective effect between 25 (OH) D and breast cancer in both cohort studies (RR = 0.85, 95% CI:0.74– 0.98) and case-control studies (OR = 0.65, 95% CI: 0.56–0.76). However, analyzing by menopausal status, the protective vitamin D – breast cancer association persisted only in the pre-menopausal group (OR = 0.67, 95% CI: 0.49–0.92) when restricting the analysis to nested case-control studies. No significant association was found for vitamin D intake or 1,25(OH)2D.	This systematic review suggests a protective relationship between circulating vitamin D (measured as 25(OH) D) and breast cancer development in pre-menopausal women.	The number of studies is still limited and publication biases cannot be excluded.	Each article uses different cutoff points according to serum levels of vitamin D.<br><br>There is huge variability in the literature on the type of vitamin D studied, which makes it difficult to perform the analysis.<br><br>Levels of vitamin D depend on the season.<br><br>Case-control studies are more prone to methodological issues.<br><br>Breast cancer is a heterogeneous disease and it is possible that vitamin D only affects certain breast cancer subtypes	5/7
